# The landscape of molecular chaperones across human tissues reveals a layered architecture of core and variable chaperones

**DOI:** 10.1038/s41467-021-22369-9

**Published:** 2021-04-12

**Authors:** Netta Shemesh, Juman Jubran, Shiran Dror, Eyal Simonovsky, Omer Basha, Chanan Argov, Idan Hekselman, Mehtap Abu-Qarn, Ekaterina Vinogradov, Omry Mauer, Tatiana Tiago, Serena Carra, Anat Ben-Zvi, Esti Yeger-Lotem

**Affiliations:** 1 Department of Clinical Biochemistry and Pharmacology and the National Institute for Biotechnology in the Negev, Ben-Gurion University of the Negev, Beer Sheva, Israel; 2 Department of Life Sciences, Ben-Gurion University of the Negev, Beer Sheva, Israel; 3 Centre for Neuroscience and Nanotechnology, Department of Biomedical, Metabolic and Neural Sciences, University of Modena and Reggio Emilia, Modena, Italy

**Keywords:** Proteome, Chaperones, Data integration, Systems analysis

## Abstract

The sensitivity of the protein-folding environment to chaperone disruption can be highly tissue-specific. Yet, the organization of the chaperone system across physiological human tissues has received little attention. Through computational analyses of large-scale tissue transcriptomes, we unveil that the chaperone system is composed of core elements that are uniformly expressed across tissues, and variable elements that are differentially expressed to fit with tissue-specific requirements. We demonstrate via a proteomic analysis that the muscle-specific signature is functional and conserved. Core chaperones are significantly more abundant across tissues and more important for cell survival than variable chaperones. Together with variable chaperones, they form tissue-specific functional networks. Analysis of human organ development and aging brain transcriptomes reveals that these functional networks are established in development and decline with age. In this work, we expand the known functional organization of de novo versus stress-inducible eukaryotic chaperones into a layered core-variable architecture in multi-cellular organisms.

## Abstract (web summary)

Tissue-specific differences in protein folding capacities are poorly understood. Here, the authors show that the human chaperone system consists of ubiquitous core chaperones and tissue-specific variable chaperones, perturbation of which leads to tissue-specific phenotypes.

## Introduction

Chaperones are highly conserved molecular machines that control cellular protein homeostasis (proteostasis). Across species, they promote de novo protein folding and protein maturation^1^, protein translocation^2^, protein-complexes assembly and disassembly^3^, protein disaggregation and refolding^4^, and protein degradation^5^. In accordance with their fundamental roles, chaperones are abundant proteins. In human cell lines, for example, they were shown to compose ~10% of the total proteome mass^6^.

Chaperones have been grouped into families based on their molecular mass, common domains, protein structure similarity, and common function^1^. Families composing the main chaperone machinery, which modulate protein structure without participating in the final protein complex, include prefoldin^7^, the small heat shock proteins (sHSP)^8^, and the main ATP-hydrolyzing chaperones, HSP60^9^, HSP70^10^, HSP90^11^, and HSP100^12^. Families of co-chaperones modulate the activity of main chaperones by regulating their ATPase cycle or the recognition, binding, or release of chaperone substrates, and include HSP10^9^, HSP40 (DNAJ)^13^, nuclear exchange factors (NEFs)^14^, and co-HSP90^15^. Folding enzymes that catalyze folding-accelerating reactions, such as peptidyl prolyl *cis*–*trans* isomerization or protein disulfide isomerization^16,17^, are also considered as chaperones.

The chaperone system is highly versatile. Most chaperones interact with multiple co-chaperones, and co-chaperones can interact with multiple chaperones, thereby modifying their function or the fate of their substrates. For example, the same HSP70 chaperone can interact with different HSP40 co-chaperones, altering its substrate specificity^13^. Likewise, co-chaperones containing the TPR domain, such as Hop, interact with both HSP70 and HSP90, thereby targeting substrates for either folding or degradation, respectively^18^. The versatility and robustness of the chaperone system manifests in stress conditions, which lead to extensive upregulation of some chaperones. However, the system also has limitations. Whereas chaperone overexpression typically improves the folding capacity of the chaperone system, overexpression of specific chaperones was shown to disrupt folding^19–23^. For example, overexpression of the folding enzyme FKBP51 in a tau transgenic mouse model resulted in accumulation of tau and its toxic oligomers^21^. Likewise, chaperone downregulation or genetic aberration can cause diseases, such as mutation in the NEF co-chaperone BAG3 that leads to myopathy^24^. These observations imply that the quantitative composition of the chaperone system can improve or impair its proteostatic capacity.

The chaperone system has expanded considerably in evolution^25,26^. The HSP40 family, for example, expanded from three members in *E. coli* and 22 members in budding yeast to 49 members in human, whose distinct functionalities are not totally clear^13^. The evolution of the sHSP family is more complex, as plants and some lower eukaryotes have more sHSPs than mammalian and higher eukaryotes^27^. Consequently, the chaperone system has been remodeled. Whereas in prokaryotes the same chaperones carry both de novo protein folding and response to stress, unicellular eukaryotes evolved two separately regulated chaperone systems, a basal system and a stress inducible system, each composed of distinct members of the same chaperone families^28^. This two-level organization is conserved in multi-cellular eukaryotes. Yet, multi-cellular eukaryotes are also composed of multiple cell types, tissues, and organs, each having different proteomes and thus potentially different folding demands. For example, HSPB1 is required for actin and myofibril assembly and its depletion specifically impairs cardiac progenitor fusion and heart tube formation^29^.

Recent years were marked by multiple large-scale mappings of the proteomic and transcriptomic landscapes of tens of human tissues. Major efforts included the Human Protein Atlas (HPA)^30^, Fantom5^31^, and the Genotype Tissue Expression (GTEx) consortium^32^. These resources enabled unprecedented quantitative views into the genes and proteins that make up physiological human tissues. These studies and others have revealed tissue-specific regulatory elements, molecular interaction networks, and functional mechanisms underlying traits and diseases^32–36^. Large-scale studies of the chaperone system were performed in the context of neurodegenerative diseases^37^, heat stress^38^, and cancer^39^ via analysis of samples gathered from patients. These studies revealed different patterns of chaperone network dysregulation. However, a systematic examination of the basal chaperone network in physiological human tissues has been lacking.

In this work, we harness transcriptomic profiles gathered by the GTEx consortium^32^, as well as other publicly available omic datasets^30,37,40–43^, to systematically examine the basal chaperone system in various human tissues. We focus on 194 manually curated chaperones, co-chaperones, and folding enzymes of the main chaperone families, henceforth collectively referred to as chaperones. We find that in accordance with the fundamental role of the chaperone system, chaperones are significantly more ubiquitously and highly expressed across all tissues relative to other protein-coding genes, and are also more important for growth. Nevertheless, differential analysis of chaperone expression across tissues shows that most chaperones have tissue-specific behaviors. A proteomic screen of mouse myoblasts cell line and a computational analysis of chaperones with a known aberration that is causal for a Mendelian disease show that these tissue-specific behaviors tend to be conserved and functional. For the community interested in specific chaperones we present a website, https://netbio.bgu.ac.il/chapnet/, for browsing the information on specific chaperones and their relationships across tissues. We further highlight a core set of chaperones that is uniformly expressed across tissues, and show them to be more important for growth and more highly expressed than other chaperones. This core subsystem establishes tissue-specific functional networks, which are enhanced by their tissue-specific relationships with other chaperones. Notably, by analyzing transcriptomic datasets of human organ development^42^ and brain aging^37^, we find that the core versus variable organization and the tissue-specific functional networks are established throughout development and are challenged in aging. We propose that the combination of core and non-core chaperones constitutes a third-level organization of the chaperone system that is capable of supporting the different proteostatic demands of multi-cellular eukaryotes, and that this organization illuminates the phenotypic outcomes of chaperone aberrations.

## Results

### Chaperones are ubiquitously and highly expressed across human tissues

We manually curated a list of chaperones and co-chaperones of the main eukaryotic chaperone families, which drive basic cellular processes needed in living cells. These included sHSP, HSP40, HSP60/HSP10, HSP70, HSP90, prefoldin, and folding enzymes, as well as chaperones not structurally or functionally associated with a specific chaperone family, such as ER chaperones, which we denoted as “other” (see the “Methods” section, Supplementary Data 1). Most of the chaperones in this list were not substrate-specific. We also included the myosin chaperone UNC45 and the collagen chaperone SERPINH1, since they interact with multiple members of the myosin and collagen protein families, respectively (Supplementary Data 1). To analyze chaperone expression across tissues, we used RNA-sequencing profiles of adult human tissues that were made available by the GTEx consortium^32^. We focused on tissues with at least five available profiles sampled from donors with traumatic injury fitting with sudden death, and avoided other death causes to limit the effect of treatment, inflammation, and stress on chaperone expression^44–47^. This resulted in a total of 29 tissues, 488 profiles, and 194 expressed chaperones, co-chaperones and folding enzymes, collectively referred to as chaperones (Supplementary Data 1 and 2).

**Fig. 1:**
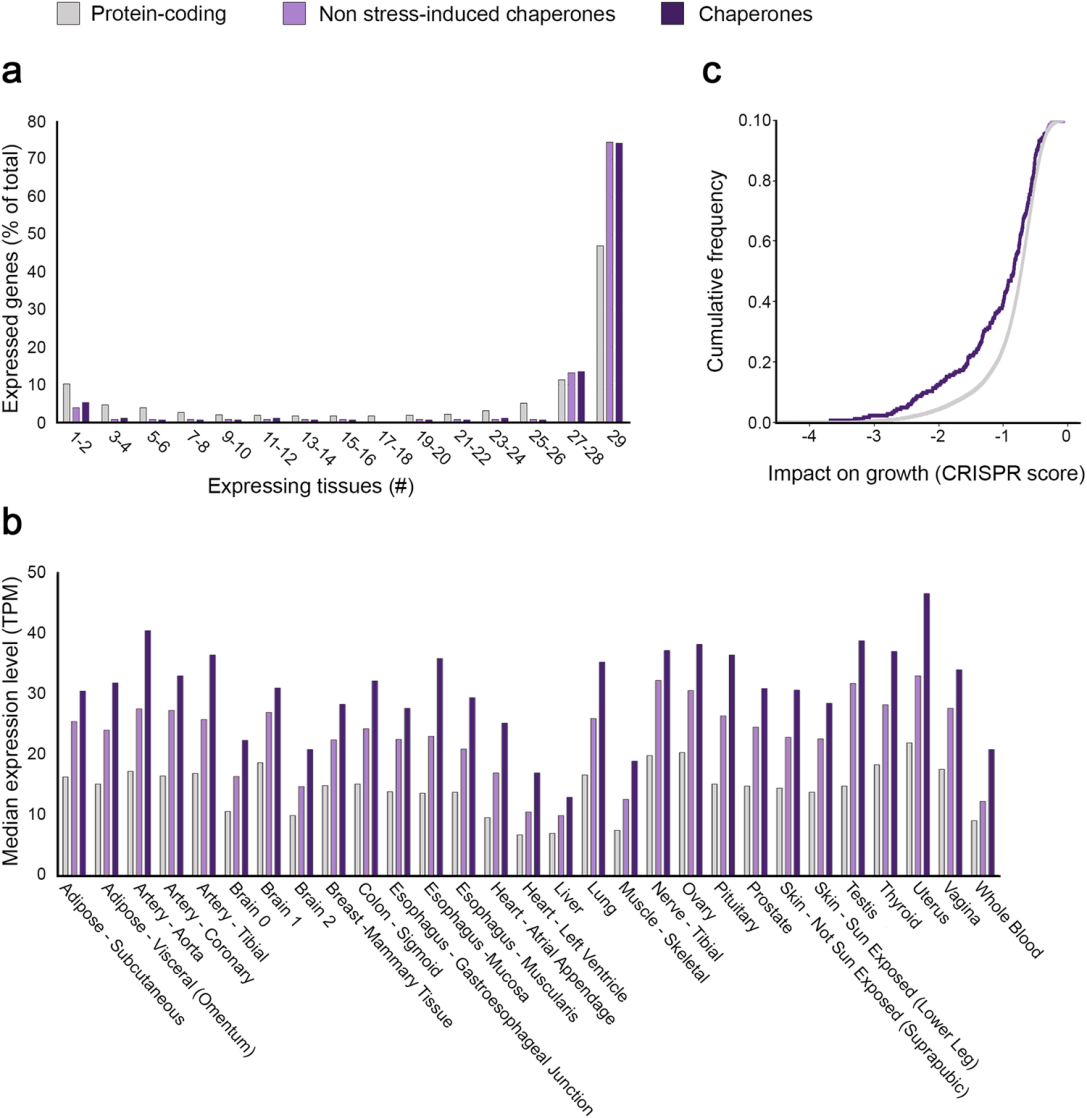
Chaperones are broadly expressed across human tissues. **a** The distribution of 194 chaperones, 130 non-stress-induced chaperones and 17,689 other protein-coding genes by the number of tissues expressing them at a level ≥1 transcripts per million (TPM; the first bin represents genes expressed in a single tissue or two tissues, the second bin represents genes expressed in three or four tissues, etc.). Chaperones and non-stress-induced chaperones were significantly more broadly expressed than other protein-coding genes (*p* = 2.7E−9 for both, two-sided Kolmogorov–Smirnov test). **b** The median expression levels per tissue of chaperones, non-stress-induced chaperones and other protein-coding genes. Only genes expressed at a level ≥1 TPM were considered. Chaperones and non-stress-induced chaperones tend to be significantly more highly expressed across all 29 tissues (adjusted *p* between 1.7E−7 and 1.4E−19, and adjusted *p* between 0.026 and 4.9E−8, respectively, two-sided Mann–Whitney test). Chaperones *n* = 155–192; non-stress-induced chaperones *n* = 102–129; other protein-coding genes *n* = 10,689–15,555. **c** The cumulative distribution of the impact on growth of 186 chaperones and 17,932 other protein-coding genes, measured in 769 cell lines harboring CRISPR-induced gene inactivation (CRISPR score). Each gene was associated with its minimal CRISPR score. Chaperones were significantly more important for growth than other protein-coding genes (*p* = 1.5E−5, one-sided Kolmogorov–Smirnov test). Source data are provided as a Source Data file.

We first compared the distribution across tissues of expressed chaperones to that of other protein-coding genes. We considered a gene as expressed in a tissue if its median expression level in samples of that tissue was above commonly used expression thresholds (1, 5, or 10 transcripts per million (TPM), see the “Methods” section). The number of chaperones expressed in each tissue at a threshold of 1 TPM was similar (176 ± 6, Supplementary Data 2). Next, we associated each gene with the number of tissues in which it was expressed (Fig. 1a, Supplementary Fig. 1A). This resulted in a bi-modal distribution, as previously shown for protein-coding genes^48^. However, chaperones were significantly more broadly expressed than other protein-coding genes (*p* = 2.7E−9, Kolmogorov–Smirnov (KS) test, Fig. 1a). For example, 74% of the chaperones were expressed in all tissues, relative to 47% of the other protein-coding genes. Similar results were observed with different TPM thresholds (Supplementary Fig. 1A).

Next, we examined the expression levels of chaperones relative to other protein-coding genes. Within each of the 29 tissues that we analyzed, the expression levels of chaperones were significantly higher (*p* < 1.7E−7, Mann–Whitney (MW) test, Fig. 1b), also upon considering other TPM thresholds (Supplementary Fig. 1B). Since some chaperones are known to be upregulated in stressful conditions, they could be driving the high expression that we observed. To test for this, we defined a subset of 64 stress-induced human chaperones, which were determined previously by using a meta-analysis of microarray data^38^ (see the “Methods” section). Indeed, stress-induced chaperones were more highly expressed than non-stress-induced chaperones (Supplementary Fig. 1C). Nevertheless, upon excluding stress-induced chaperones from the analysis, the remaining non-stress-induced chaperones were still significantly more broadly expressed (*p* = 2.7E−9, Fig. 1a and Supplementary Fig. 1A) and more highly expressed than other protein-coding genes (*p* < 0.026, Fig. 1b and Supplementary Fig. 1B). Finally, to validate the generality of these observations, we repeated the above analyses by using transcriptomic profiles of 37 human tissues that were made available by the HPA^30^. The results obtained with this dataset were similar to those obtained with the GTEx dataset, reinforcing our conclusions (Supplementary Figs. 2A–C and 3).

To further examine to what extent chaperones are fundamental components of living cells, we utilized large-scale data of gene essentiality from the DepMap project^41^. The DepMap dataset measured the impact of CRISPR-induced individual gene inactivation on growth rate in 769 human cell lines (see the “Methods” section). We found that relative to inactivation of other protein-coding genes, inactivated chaperones lowered growth rates significantly, and were thus considered more important for growth (*p* = 1.5E−5, KS test, Fig. 1c). The relatively high impact of chaperones could be common to other ubiquitously expressed protein-coding genes. However, upon limiting our analysis to the subsets of chaperones and other protein-coding genes that were ubiquitously expressed, chaperones were still more important for growth (*p* = 0.0025, KS test, Supplementary Fig. 1D). Similar results were obtained by using gene essentiality scores in 318 cell lines from Project Score^40^ (Supplementary Fig. 4A, B). Altogether, chaperones appear to constitute a ubiquitous and highly expressed cellular system that is fundamental for cell growth.

### Chaperone expression levels vary across human tissues

**Fig. 2:**
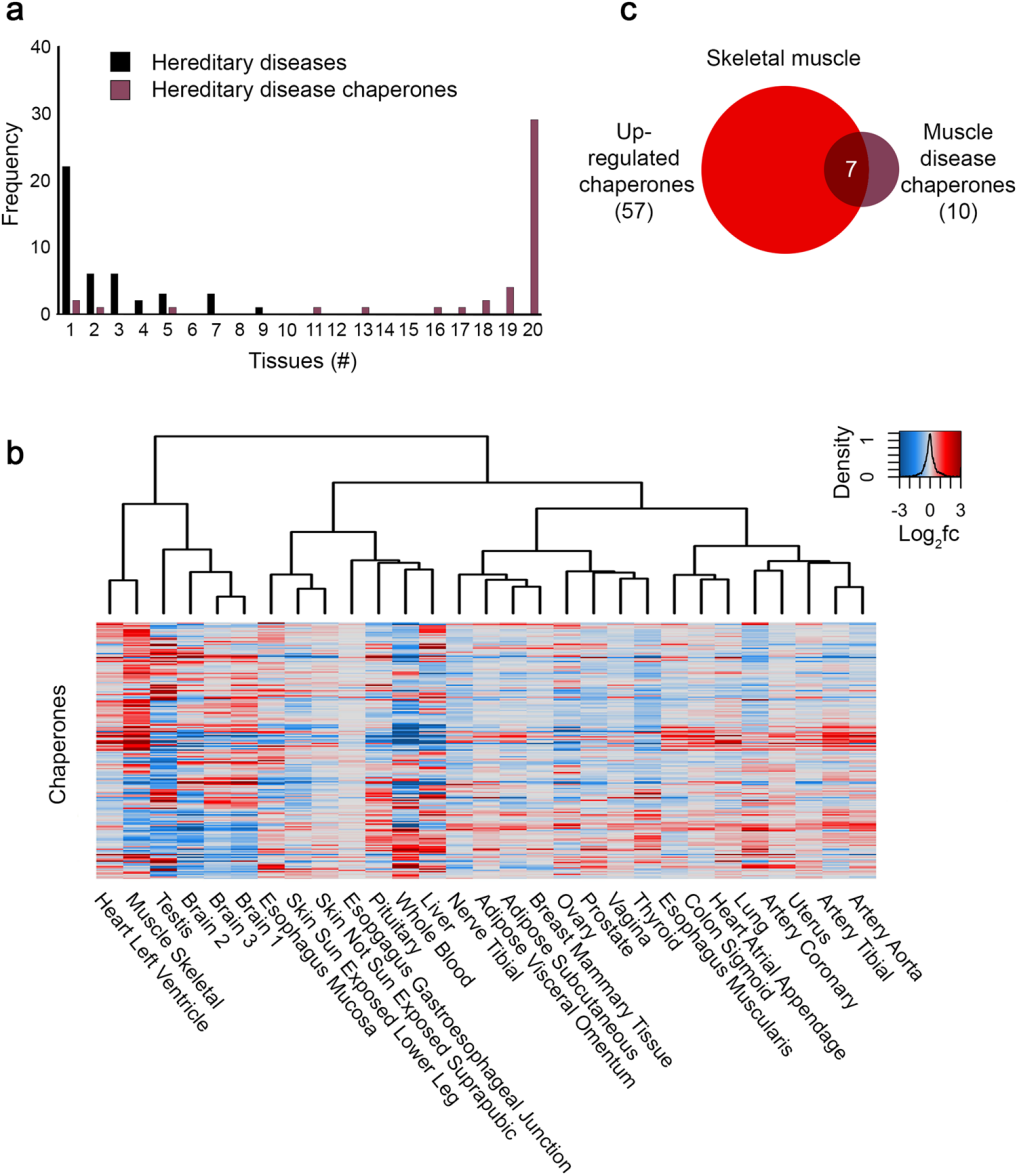
The variable expression of chaperones across human tissues. **a** The distribution of 43 chaperones with known aberrations that are causal for Mendelian diseases by the number of tissues expressing them at a level ≥1 transcripts per million (TPM) or above (denoted heredity disease chaperones), and the distribution of the respective 62 Mendelian diseases by the number of tissues in which they manifest clinically (denoted heredity diseases). We united sub-parts of the same tissue (e.g., adipose subcutaneous and adipose visceral omentum were united into a single adipose tissue), ending up with 20 united tissues. Most hereditary diseases are highly tissue-specific (Supplementary Data 3). In contrast, hereditary disease chaperones are expressed ubiquitously across tissues, also when considering other expression thresholds (Supplementary Fig. 1E). **b** A clustered heatmap showing the differential expression of 194 chaperones across tissues. Differential expression of a chaperone in a tissue was computed by comparing the expression profiles of that tissue to the expression profiles of all other tissues. Each entry reflects the log_2_ fold-change value (log_2_fc) of a chaperone (row) in a tissue (column); red and blue denote positive and negative log_2_fc values, respectively. Physiologically related tissues often clustered together. **c** The overlap between 57 chaperones that were upregulated (log_2_fc ≥ 1) in human skeletal muscle relative to other tissues, and the 10 chaperones with a known aberration that causes heritable muscle disorders. Seven chaperones were both upregulated and associated with muscle disorders (*p* = 0.0078, one-sided Fisher exact test). Source data are provided as a Source Data file.

Despite the ‘house-keeping’ functionality of chaperones, different tissues have different folding, assembly, and maintenance demands. For example, the demands of the sarcomere in muscle cells, which contain titin, the largest protein in the human body^49,50^ likely vary from those of synaptic neurotransmission in neurons, where the membrane protein synaptic vesicle protein 2 (SV2) is amongst the most abundant and conserved components^51,52^. Such demands become phenotypically apparent in face of mutations. To test whether these demands are met by tissue-specific chaperones, we gathered 62 tissue-selective heritable disorders known to be caused by aberrations in 43 distinct chaperones^34,53,54^, and analyzed the expression patterns of these chaperones across tissues (see the “Methods” section, Supplementary Data 3). In contrast to the tissue-specific clinical manifestation of these disorders, most of the 43 underlying chaperones were expressed ubiquitously across tissues (Fig. 2a), also when considering other expression thresholds (Supplementary Fig. 1E), in agreement with the behavior of other tissue-selective heritable disorders^55,56^. Thus, chaperone disruption is harmful only in specific tissues, likely depending on tissue folding demands.

Next, we asked whether the expression levels of each chaperone remain high and uniform, or whether they vary across tissues. For this, we computed the differential expression of chaperones in any given tissue relative to all other tissues^57^ (see the “Methods”” section, Supplementary Data 4). Most chaperones (162, 83.5%) had over 2-fold change in their expression in at least one tissue, henceforth considered as variable chaperones. To display the expression variability of chaperones across tissues, we clustered chaperones and tissues hierarchically according to their differential expression profiles (Fig. 2b). Physiologically related tissues, such as skeletal muscle and heart, or different brain tissues, clustered together, suggesting a biological relevance to the differential expression patterns of chaperones.

To explore the biological relevance of tissue-specific differential expression profiles, we asked whether tissue-specific changes in expression were related to tissue-specific phenotypes. We focused on skeletal muscle, since it showed a distinct differential expression profile relative to other tissues. Notably, this distinct profile was not due to a higher number of expressed chaperones in skeletal muscle relative to other tissues (170 versus average of 176 chaperones, Supplementary Data 2), or due to a high total expression of chaperones (11,604 TPMs versus average of 14,283 TPMs).

We found that out of the 10 chaperones in which mutations that cause disorders affecting skeletal muscle were identified (Supplementary Data 3), seven chaperones were expressed at least 2-fold higher in skeletal muscle compared to other tissues (*p* = 0.0078, Fisher exact test, Fig. 2c). For example, DNAJB6 [log_2_ fold-change (log2FC) of 2.24] and the NEF chaperone BAG3 (log2FC of 3.13) lead to Limb-girdle muscular dystrophy 1E^58^ and myopathies, respectively^59–61^. Mutations in the three remaining chaperones (BCS1L, SIL1, and FKBP14) lead to diseases that affect multiple tissues apart from skeletal muscle (Supplementary Data 3). We also examined the remaining chaperones that were upregulated in skeletal muscle for association with muscle disease or function by literature mining. We found evidence for 31/50 chaperones (Supplementary Table 1), including 17 chaperones that were associated with muscle diseases (e.g., HSPB8 with myopathies) and 27 chaperones that were associated with muscle function (e.g., the myosin chaperone UNC45B). Thus, chaperone expression levels are likely designed to meet tissue-specific demands, and consequently affect disease risks in a tissue-specific manner.

### A conserved chaperone expression pattern in muscle

**Fig. 3:**
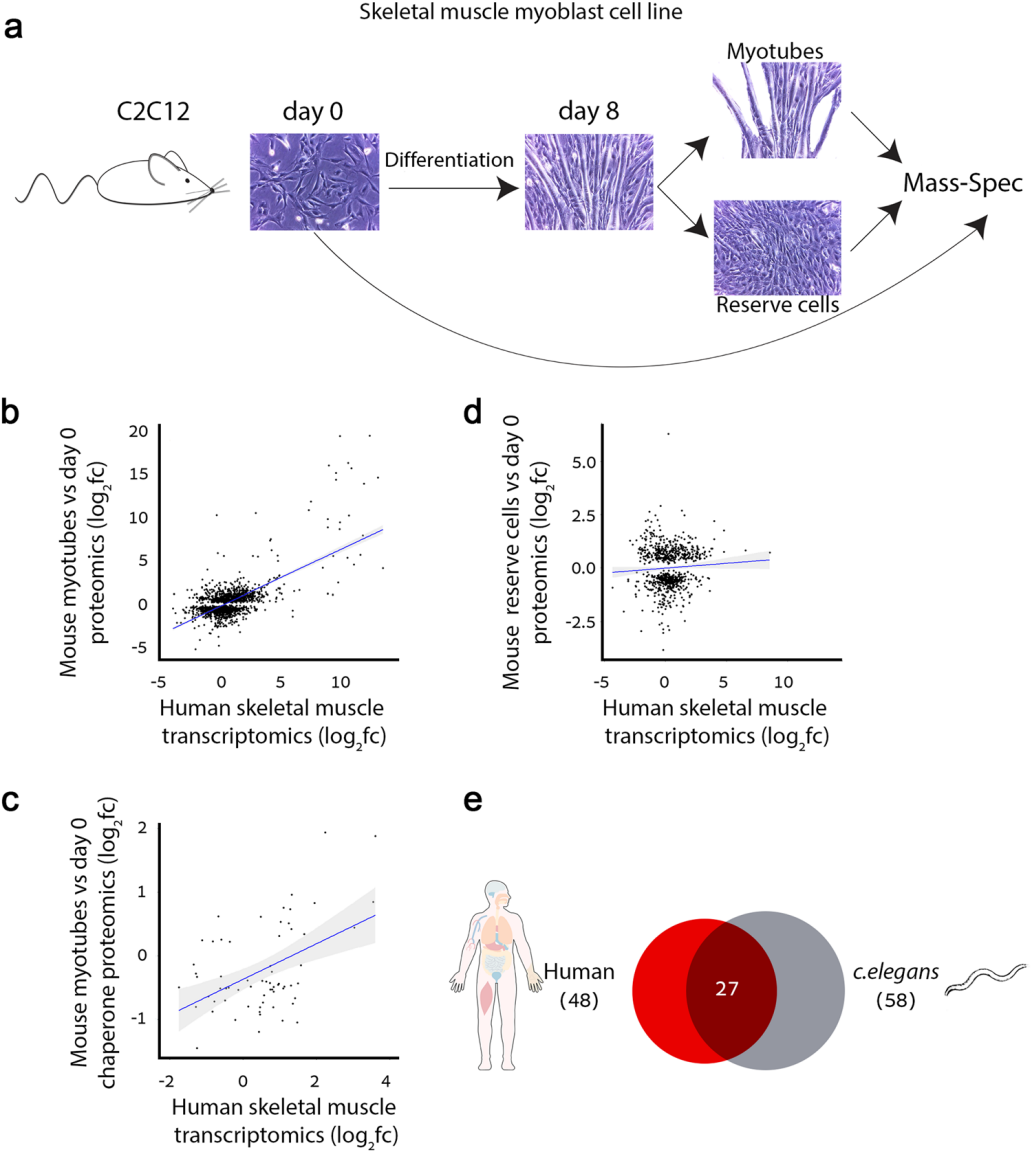
Chaperone expression in muscle tissue is evolutionary conserved. **a** The experimental pipeline. C2C12 mouse myoblast cell line was grown to 95% confluency and differentiation was induced. After 8 days of differentiation, cells were separated to myotubes and reserve (undifferentiated) cells, and their proteomes were analyzed using mass spectrometry (three biological replicates for each treatment were used). **b** The correlation between the differential protein levels of 1561 proteins that were reliably measured in mouse myotubes vs. undifferentiated C2C12 cells, and the differential expression of their homologous genes in human skeletal muscle (*r* = 0.65, *p* = 2.2E−16, Pearson correlation). **c** The correlation between the differential protein levels of 65 chaperones that were reliably measured in mouse myotubes versus undifferentiated C2C12 cells, and the differential expression of their homologous genes in human skeletal muscle (*r* = 0.47, *p* = 7.8E−5, Pearson correlation). **d** The differential protein levels of 754 proteins that were reliably measured in mouse reserve cells versus undifferentiated cells do not correlate with the differential expression of their homologous genes in human skeletal muscle (*r* = 0.06, Pearson correlation). **e** The overlap between 58 *C. elegans* muscle chaperones and 48 homologous chaperones that were up-regulated (log_2_fc ≥ 1) in human skeletal muscle (*p* = 0.0009, one-sided Fisher exact test). The 157 human chaperones with homologous genes in *C. elegans* were considered in the analysis. Source data are provided as a Source Data file.

Since our analyses relied on transcriptomic datasets, which might not be informative of tissue proteomes, we decided to test chaperone expression patterns at the protein level. We first compared the transcriptomic profile of skeletal muscle to a recently published proteomic profile of the same tissue^43^. The two profiles correlated significantly for chaperones and protein-coding genes (*r* = 0.61 and *r* = 0.37, respectively, *p* = 2.2E−16, Pearson correlation, Supplementary Fig. 5A). Next, we asked whether this pattern is conserved. For this, we profiled the proteome of the C2C12 mouse myoblast cell line before differentiation (day 0) and after 8 days of differentiation to myotubes (day 8, myotubes). A subset of undifferentiated muscle cells (reserve cells) was used as a control (day 8, reserve cells; Fig. 3a). We then compared the differential proteomic profile of C2C12 cell line (day 8 myotubes vs. day 0) to the differential transcriptomic profile of human skeletal muscle. In support of the functional relevance and conservation of the GTEx muscle transcriptomic profile, the proteomic and transcriptomic profiles were indeed correlated, both upon considering all protein-coding genes (*r* = 0.65, *p* = 2.2E−16, Pearson correlation, Fig. 3b) and upon considering chaperones alone (*r* = 0.47, *p* = 7.8E−5, Pearson correlation, Fig. 3c). In contrast, no correlation was observed when we compared the differential proteomic profile of reserve cells to the differential transcriptomic profile of human skeletal muscle (Fig. 3d).

To substantiate the experimental results, we also compared the proteomic profile of C2C12 myotubes to the transcriptomic profile of skeletal muscle from the HPA^30^ (Supplementary Fig. 5B), and to the proteomic profile of human skeletal muscle^43^ (Supplementary Fig. 5C). The proteomic profile of C2C12 myotubes indeed correlated with both datasets, though to a lesser extent (Supplementary Fig. 5B, C). Lastly, we analyzed the expression of a subset of chaperones (10 sHSP genes) using qPCR in an alternative muscle cell line model consisting of cycling versus 5 days differentiated human myoblasts (LHCNM2 cells^62^). Six of the seven sHSP that were expressed at least 2-fold higher in skeletal muscle according to the GTEx dataset were also upregulated in differentiated LHCNM2 cells (Supplementary Fig. 5D). The three remaining sHSP chaperones, which were not upregulated in skeletal muscle according to the GTEx dataset, were also not upregulated in differentiated LHCNM2 cells (Supplementary Fig. 5D). These data provide further experimental support for the conservation of the muscle-specific chaperone expression pattern.

We further tested the functional conservation of muscle chaperones by comparing between human and the evolutionary distant multicellular organism *Caenorhabditis elegans*. We used a set of *C. elegans* muscle chaperones^19^, and compared them to human chaperones with *C. elegans* orthologs that were expressed at least 2-fold higher in skeletal muscle relative to other tissues (see the “Methods” section, Supplementary Table 1). We found that the two subsets overlapped significantly (27 muscle chaperones, *p* = 0.0009, Fisher exact test, Fig. 3e). For example, the HSP40 protein DNAJB6 had over 4-fold change in human skeletal muscle and its *C. elegans* homolog, *dnj-24*, was highly expressed in *C. elegans* body-wall muscle cells. Moreover, 23/27 orthologous muscle chaperones had a known association with muscle folding, such as the myosin chaperone UNC45, and the CCT subunits that are linked to actin folding (Supplementary Table 1). Altogether, these observations suggest an inherent requirement for chaperones in muscle tissue function that is conserved in evolution.

### Chaperone families show variable behavior across tissues

Since members of each chaperone family share functional and structural attributes, we examined whether they also share expression patterns. We focused on the tendency of chaperones for ubiquitous expression, for variable expression across tissues, and their impact on growth. To assess the ubiquitous expression of each chaperone family, we recorded the number of tissues in which chaperones were expressed (as in Fig. 1a, Supplementary Data 1). Apart from sHSPs, all families were more ubiquitously expressed than the rest of the protein-coding genes (adjusted *p* < 0.029, MW test). Members of the Prefoldin family and the ATP-hydrolyzing chaperone families were ubiquitously expressed, except for the HSP60 chaperonin that encodes a CCT subunit-like protein, CCT8L2, which was expressed specifically in testis (Supplementary Fig. 6A). Members of co-chaperone families showed a more variable pattern, with both tissue-specific and ubiquitously expressed members, except for NEFs (Supplementary Fig. 6A). These included, for example, the skeletal muscle-specific coHSP90 chaperone UNC45B. sHSPs were more tissue-specific than other chaperone families (adjusted *p* = 0.027, MW test). In fact, sHSPs were also more commonly causal for tissue-specific and neuro-muscular diseases^63–68^ (5/10 sHSPs, Supplementary Data 3).

To assess the tendency of chaperone families for uniform vs. variable expression across tissues, we associated each chaperone with the number of tissues in which it was variably expressed (Supplementary Fig. 6B, Supplementary Data 1). All families, except HSP70s and sHSPs, were expressed more uniformly across tissues than the rest of the protein-coding genes (adjusted *p* < 0.018, MW test). HSP60s and coHSP90s were also expressed more uniformly than other chaperone families (adjusted *p* ≤ 0.019, MW test). In contrast, sHSPs and HSP70s were more variably expressed than other families (adjusted *p* ≤ 0.033, MW test), with sHSPs also more variably expressed than other protein-coding genes (adjusted *p* = 0.015, MW test). Notably, HSP70 essential members, including the main cytosolic member HSPA8, the ribosome-associated member HSPA14, the ER member HSPA5 and the mitochondrial member HSPA9, were relatively uniformly expressed (Supplementary Fig. 6B, Supplementary Data 1).

Lastly, we assessed the impact on growth of each family (Supplementary Fig. 6C, Supplementary Data 1). Prefoldins, HSP60s, HSP70s, and coHSP90s were more important for growth than the rest of the protein-coding genes (adjusted *p* < 0.05, MW test), and prefoldins and HSP60s were also more important for growth than other families (adjusted *p* ≤ 0.045, MW test). However, the impact of individual chaperones on growth was highly diverse in most chaperone families. For example, most CCT subunits were important for growth, yet CCT subunits such as the testis-specific CCT8L2 and the variably expressed CCT6B were less important. sHSPs were less important for growth than other chaperone families (adjusted *p* < 0.066, MW test), fitting with them having tissue-specific expression. In summary, whereas certain features were family-based, members of most chaperone families showed distinct features hinting to distinct functionalities across tissues.

### Core chaperones versus variable chaperones

**Fig. 4:**
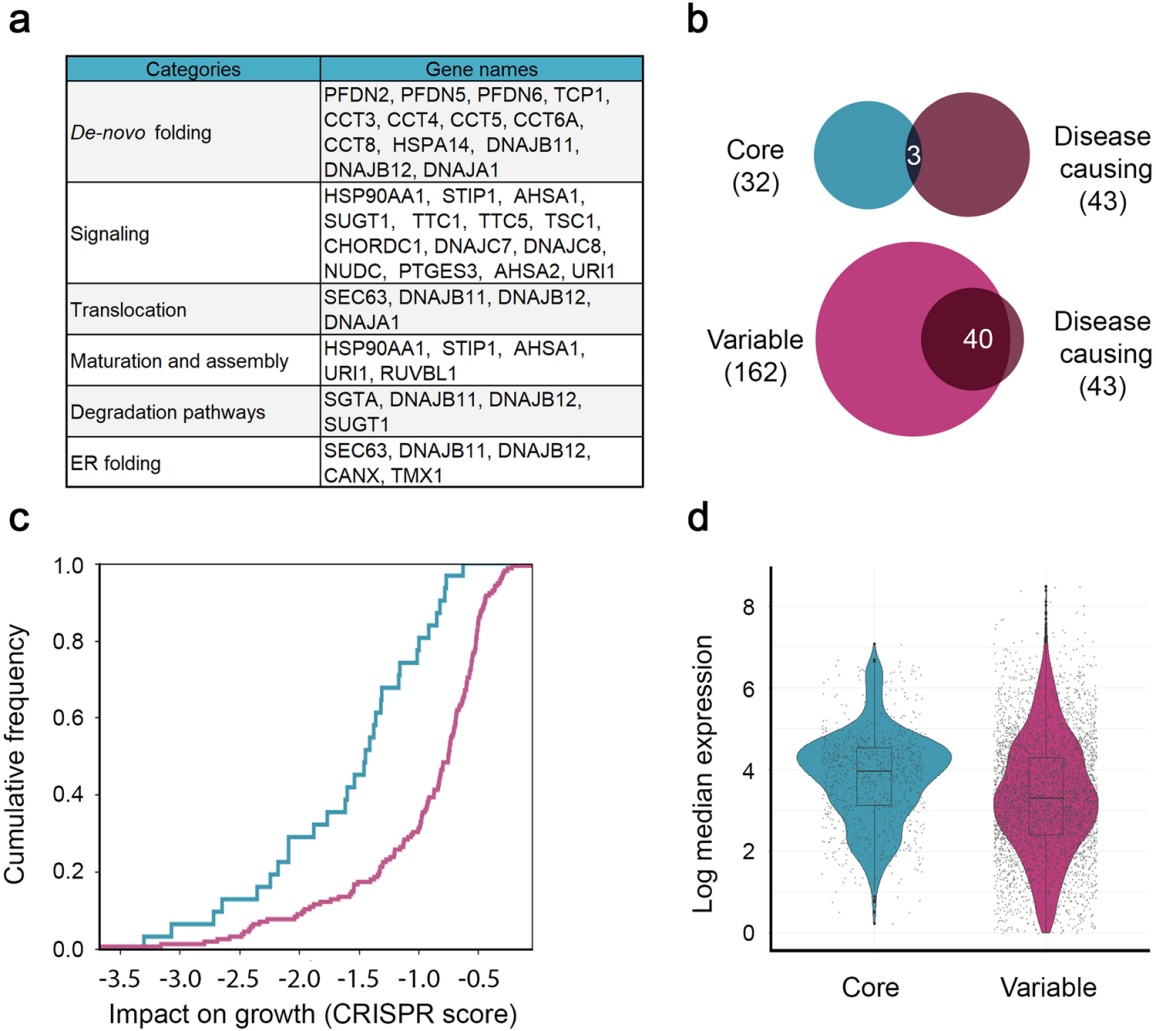
The fundamental roles and impact of core chaperones. **a** The functional roles of the 32 core chaperones. Chaperones with several functions appear in all categories that apply. Core chaperones performed basic function required by different cell types. **b** The overlap between core chaperones (top) or variable chaperones (bottom), and chaperones with known aberrations that are causal for Mendelian diseases. Core chaperones tend to be depleted of such chaperones, whereas variable chaperones tend to be enriched for them (*p* = 0.04, one-sided Fisher exact test). **c** The cumulative distribution of the impact on growth of core (blue) versus variable chaperones (pink) measured in 769 cell lines harboring CRISPR-induced gene inactivation. 31 core and 155 variable chaperones for which CRISPR scores were available were considered. Each gene was associated with its minimal CRISPR score. Core chaperones were significantly more important for growth (*p* = 1.9E−6, one-sided Kolmogorov–Smirnov test). **d** The median expression levels per tissue of core versus variable chaperones (based on *n* = 928 and *n* = 4173 values, respectively). Core chaperones were significantly more highly expressed (*p* = 2.2E−16, one-sided Mann–Whitney test). In the boxplot representation, center line, median; box limits, upper and lower quartiles; whiskers, 1.5× interquartile range. Source data are provided as a Source Data file.

We next focused on the small subset of 32 (16%) chaperones that were expressed uniformly across tissues, henceforth denoted as core chaperones. Literature mining of the cellular processes in which these core chaperones participate showed that they covered a variety of basic processes, including de novo protein folding maturation and assembly; ER-associated targeting, transport, folding, and degradation; and translocation into the mitochondria (Fig. 4a). Core chaperones were also slightly enriched for Prefoldins, CCTs, and coHSP90s, and were depleted for sHSPs.

Next, we asked whether core chaperones included chaperones with known heritable disease-causing aberrations (see the “Methods” section). Only 3/32 core chaperones had such aberrations, in contrast to variable chaperones, which were significantly enriched for them (*p* = 0.04, Fisher exact test, Fig. 4b). This suggests that core chaperones do not cause diseases, either because core chaperones are functionally redundant, or, inversely, because their aberration is embryonically lethal. One such example is the CCT5 CRISPR knockout (Cct5^em1(IMPC)Tcp^) mutant mice that showed preweaning lethality. To quantitatively distinguish between the two alternatives, we compared between the impact on growth of mutations in core and variable chaperones^69^. We found that core chaperones were significantly more important for growth than variable chaperones (*p* = 1.9E−6, KS test, Fig. 4c, Supplementary Fig. 4C), also upon limiting variable chaperone to those that were expressed ubiquitously (*p* = 2.7E−12). Lastly, we compared the median expression levels of core versus variable chaperones across tissues (Fig. 4d, Supplementary Fig. 2D). Core chaperones were significantly more highly expressed than variable chaperones (*p* < 2.2E−16, MW test). Taken together, we propose that core chaperones establish an essential subset of chaperones with stringent, uniform demand across tissues.

### Chaperone networks differ between tissues

**Fig. 5:**
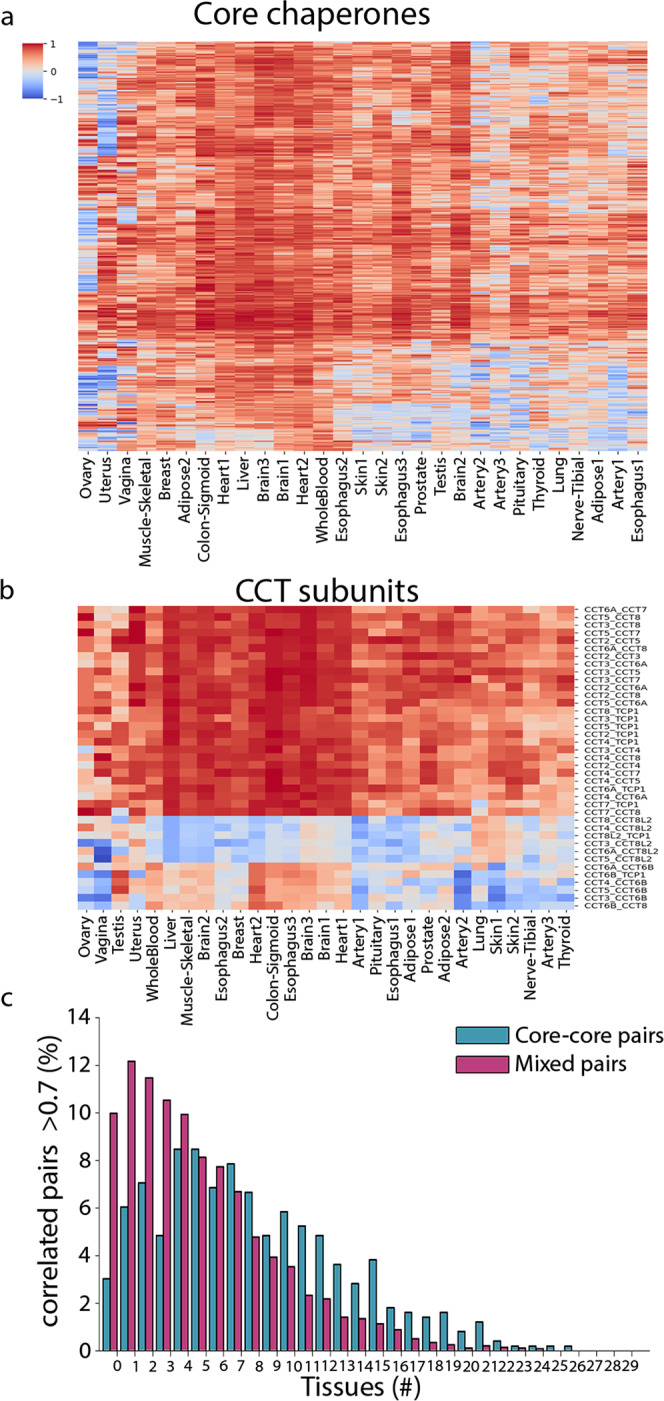
Functional relationships between chaperones differ across tissues. **a** A heatmap of the expression correlations between core chaperones. Each row corresponds to a distinct pair, and shows its expression correlation values across tissues (columns). Across tissues, core chaperones modified their co-expression relationships with each other, thereby creating tissue-specific networks with distinct folding capacities. **b** A heatmap of the expression correlations between CCT subunits. Each row corresponds to a distinct pair, and shows its expression correlation values across tissues. Mixed pairs of CCT subunits were less correlated in their expression across tissues than core pairs. **c** The distribution of pairs of core chaperones (blue) and mixed pairs of core and variable chaperones (pink) by the number of tissues in which their expression levels were correlated (*r* > 0.7, Spearman correlation). Pairs of core chaperones tend to be correlated across more tissues relative to mixed pairs (*p* = 6.2E−5, two-sided Kolmogorov–Smirnov test). Similar results were obtained using a different threshold (Supplementary Fig. 7B). Source data are provided as a Source Data file.

Chaperones act as part of a functional network. Previous studies analyzed the physical interactions of a subset of chaperones in tissue culture^20^ and cancer cell lines^70^, or studied chaperones that were co-regulated in cancer samples^39^ or in aging^37^. Yet, the functional network of chaperones in physiological human tissues was rarely analyzed. To examine this network, we focused on the co-expression of chaperone pairs across tissues. For each pair of chaperones and for each tissue, we computed the pairwise correlation between chaperone expression levels across samples of that tissue (Supplementary Data 5). We then constructed a heatmap presenting these correlations for pairs composed of core chaperones (Fig. 5a) and for mixed pairs composed of core and variable chaperones (Supplementary Fig. 7A). Though core chaperones were uniformly expressed, their expression levels were not uniformly correlated across tissues (Fig. 5a). In some tissues, such as heart and brain, pairs showed similar and relatively high correlations. However, in most other tissues, and more so among mixed pairs, the correlations varied, suggesting that chaperones create tissue-specific networks with distinct folding capacities.

To demonstrate this, we focused on the CCT complex, which is composed of core and variable subunits (Fig. 5b). Again, we found that in a subset of the tissues, a subset of CCT subunits were highly correlated. Strikingly, CCT pairs that showed low or anti-correlated expression were all mixed pairs (e.g., the variable chaperones CCT6B or CCT8L2 with core chaperones). These co-expression changes hint toward the functional plasticity of the chaperone network across tissues. For example, the core chaperone CCT5 was highly correlated with the variable chaperone CCT6B specifically in testis (*r* = 0.89), whereas in other tissues CCT5 was highly correlated with the core chaperone CCT6A, the paralog of CCT6B (median correlation 0.79; Fig. 5b). In agreement, CCT6B high levels are specifically associated with testicular cancer, whereas high levels of CCT6A are associated with a broad range of cancers^71^.

To quantify the tissue-specificity of functional relationship between core–core versus mixed chaperone pairs at large-scale, we associated each chaperone pair with the number of tissues in which the pair was highly correlated (see the “Methods” section). We found that the mixed relationships were significantly more tissue-specific than core–core relationships (*p* = 6.2E−5, KS test, Fig. 5c and Supplementary Fig. 7B), thereby giving rise to distinct, tissue-specific functional networks.

To facilitate interrogation of the functional relationships between chaperones across tissues, we created the ChaperoneNet webtool (https://netbio.bgu.ac.il/chapnet/). Users can query ChaperoneNet by chaperone and tissue, and obtain a graphical network representation of the functional relationships of the query chaperone in that tissue. The network highlights core chaperones, marks chaperone families, and reports known disease associations.

### Consistent chaperone organization across development and aging

**Fig. 6:**
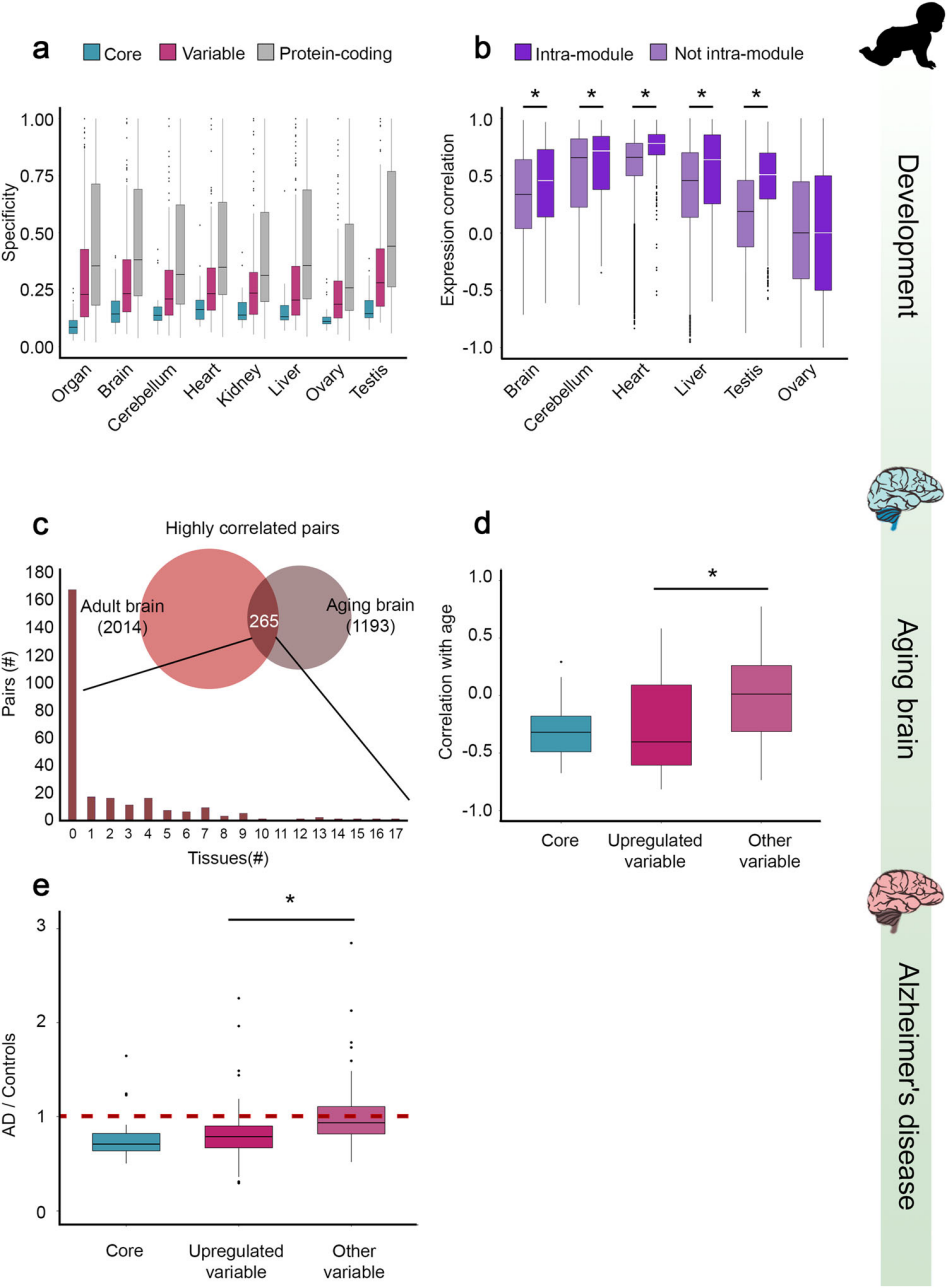
Chaperones organization is conserved in organ development and brain aging. **a** Organ specificity and organ time point specificity of protein-coding genes (16,955 genes) and chaperones (31 core and 156 variable genes). Specificity values ranged between 0 (non-specific expression) and 1 (specific expression). Across organs and time points, chaperones were more ubiquitously expressed than protein-coding genes, and core chaperones were more ubiquitously expressed than variable chaperones. Adjusted *p*-values for organ specificity and time point specificity in brain, cerebellum, heart, kidney, liver, ovary, and testis, in respective order: chaperones versus protein-coding genes: *p* = 4.8E−16, 3.24E−18, 7.26E−17, 8.16E−20, 1.48E−14, 3.2E−21, 3.27E−15, 1.9E−20; core versus variable chaperones: *p* = 5.39E−10, 4.5E−5, 1.4E−4, 2.5E−4, 1.4E−4, 6.44E−5, 3.53E−5, 1.23E−7 (one-sided Mann–Whitney test, Benjamini–Hochberg correction). Chaperones *n* = 187, 180, 171, 178, 176, 176, 177, 184; protein-coding genes *n* = 16,955, 14,517, 14,017, 13,828, 14,136, 13,854, 13,988, 15,712; core chaperones *n* = 31; variable chaperones *n* = 156, 149, 140, 147, 145, 145, 146, 153. **b** Expression correlation values in adult tissues for chaperone pairs whose pair-mates share, or do not share, a developmental module. Except for ovary, pairs belonging to the same developmental module were more highly correlated than other pairs. Adjusted *p*-values for brain, cerebellum, heart, liver, and testis, in respective order: *p* = 3.14E−14, 6.33E−8, 4.7E−79, 8.6E−27, 3.5E−231 (one sided Mann–Whitney test, Benjamini–Hochberg correction). Pairs in same module *n* = 916, 1204, 862, 694, 1818; pairs in other modules *n* = 16,851, 16,563, 16,904, 17,072, 15,948. **c** Consistency between chaperones that were highly correlated (*r* > 0.8) in adult brain (*n* = 2014 pairs) and in aging brain (*n* = 1193 pairs) is shown by their significant overlap (265 pairs, *p* = 1.4E−32, one-sided Fisher exact test). The distribution of the 265 consistent chaperone pairs by the number of non-brain tissues in which they were highly correlated reveals that most of them were brain-specific, i.e., were not highly correlated in any other tissue. **d** The correlation between chaperone expression levels in prefrontal cortex and age is shown for core chaperones (*n* = 26), variable chaperones that were upregulated in adult brain (*n* = 78), and variable chaperones that were not upregulated in brain (*n* = 73, other). Expression levels of core and brain-upregulated chaperones decreased with age. The expression levels of other variable chaperones did not change (see Supplementary Fig. 8B for additional brain regions). The difference between the two subsets of variable chaperones was statistically significant (*p* = 6.9E−6, one-sided Mann–Whitney test). **e** Relative expression levels of chaperones in patients with Alzheimer’s disease and controls is shown for core chaperones (*n* = 26), variable chaperones that were upregulated in adult brain (*n* = 78), and remaining variable chaperones (*n* = 73, other). Expression levels of core and brain-upregulated chaperones decreased in patients, whereas expression levels of remaining variable chaperones did not change. The difference between the two subsets of variable chaperones was statistically significant (*p* = 4.12E−6, one-sided Mann–Whitney test). In the boxplot representation, center line, median; box limits, upper and lower quartiles; whiskers, 1.5× interquartile range; points, outliers. Source data are provided as a Source Data file.

To understand how fundamental is the chaperone organization that we observed in adult human tissues, we went on to test whether it is maintained during human organ development. To answer this question, we relied on a recent transcriptomic analysis of seven human organs, including brain (cerebrum), cerebellum, heart, kidney, liver, ovary, and testis^42^. These organs were profiled at 23 time points that ranged from early organogenesis (14 time points in weeks 4–20 post conception) to adulthood. The study then associated each gene with measures that represented its organ specificity, and with its time point specificity in each organ (0 non-specific, 1 most specific). We first compared between the organ and time point specificities of chaperones versus other protein-coding genes. In accordance with their ubiquitous expression in adult tissues, chaperones were significantly more broadly expressed (i.e., non-specific) across developing organs and across developmental time points in each organ (*p* < 1.5E−14, MW test, Fig. 6a). Next, we tested whether the distinction between core and variable chaperones was also maintained. Indeed, relative to variable chaperones, core chaperones were significantly more broadly expressed across developing organs and across developmental time points in each organ (*p* < 2.5E−4, MW test, Fig. 6a).

As part of the transcriptomic analysis of human organs during development, genes were clustered per organ into modules according to their developmental expression patterns. We therefore asked whether chaperones that belonged to the same module in development continued to function as a module in adulthood. For this, we focused on six organs that were profiled both during development^42^ and in adulthood^32^, including brain, cerebellum, heart, liver, ovary, and testis. As shown in Fig. 6b, except for ovary, chaperone pairs belonging to the same module in development were significantly more correlated in adult tissues, relative to chaperone pairs that did not belong to the same module (*p* < 6.4E−8, MW test). Thus, chaperones belonging to the same module continue to function as a module throughout life. Interestingly, the correlations observed among core–core pairs were generally higher than those observed among mixed pairs; these, in turn, were higher than the correlations observed among variable–variable pairs, suggesting a hierarchy of functional relationships (Supplementary Fig. 8A). Taken together, these data suggest that the core versus variable organization and tissue-specific networks are consistent between developing organs and adult tissues.

It is commonly acknowledged that the deterioration observed in aging, particularly of the brain, involves reduced capacity for proper protein folding, thereby leading to increased risk for protein-misfolding neurodegenerative diseases, such as Alzheimer’s and Parkinson’s diseases^37,72^. To illuminate the relationship between aging and the brain-specific chaperone network, we relied on a large-scale analysis of chaperone expression in aging brains^37^. There, pairs of chaperones with highly correlated expression in aging brain were identified, and the expression of each chaperone was tested for its correlation with age. To examine whether the functional relationships between chaperones were conserved in aging, we compared between highly correlated pairs observed in adult brain and in aging brain. We found a highly significant overlap between them (265 pairs, *p* = 1.4E−32, Fisher exact test, Fig. 6c). Most of the overlapping pairs were highly correlated only in brain and not in other tissues (167/265 pairs, Fig. 6c). Moreover, overlapping pairs belonged primarily to modules that were repressed in aging brain^37^ (258/265). This suggests that a brain-specific chaperone network is conserved across time and is repressed with age.

Next, we focused on the main components of the brain-specific chaperone network, namely core chaperones and variable chaperones that were upregulated in adult brain, and examined the change in their expression with age. We found that the expression levels of both core chaperones and brain-upregulated variable chaperones were generally reduced with age (Fig. 6d, Supplementary Fig. 8B). In contrast, the expression levels of variable chaperones that were not upregulated in brain did not change in brain aging. This further suggests that the brain-required chaperone system declines with age. Further decline in the brain-required chaperone system was observed when we examined the relative expression levels of chaperones in Alzheimer’s disease patients versus age-matched controls^37^ (Fig. 6E). Taken together, these results suggest a reduced protein-folding capacity of a brain-specific chaperone system in aging that could contribute to age-dependent neurodegeneration.

## Discussion

Chaperones are basic components of all living organisms, and are thus commonly regarded as house-keeping genes^1^. But, are all folding environments similar to each other? In a multicellular organism, owing to the specialized structures, functions, and environments of the different cell types, folding requirements could differ greatly. This raises the question of whether the chaperone system is organized as a one size fits all, or whether the requirements of each tissue are met by a tailored system. To address this question systematically, we set out to examine the expression landscape of molecular chaperones across physiological human tissues.

We first examined the expression of chaperones across adult human tissues, as measured via RNA-sequencing by the GTEx consortium^32^. We determined that most chaperones were expressed in all tissues, were highly expressed in most of them, and were more important for growth than other protein-coding genes, suggesting that they act as major building blocks across all tissues (Fig. 1). Nevertheless, chaperones are also associated with tissue-specific phenotypes, since germline aberrations in ubiquitous chaperones or in ubiquitous aggregation-prone proteins can lead to tissue-specific pathologies (Fig. 2a), such as muscle disorders (Fig. 2c) or neurodegeneration^63,73–75^ (Fig. 6e). Indeed, we found that most chaperones were variably expressed across tissues, suggesting that the chaperone system is not simply common and consistent across tissues (Fig. 2b). We did not consider substrate-specific chaperones, whose expression likely correlates with the expression of their target (for example, SERPINH1 expression is correlated with collagen, its client, in collagen-synthesizing cells^76^). The results of our analyses are available as a webserver https://netbio.bgu.ac.il/chapnet/ that can be queried by chaperone and tissue.

The GTEx transcriptomic resource allowed for high-resolution tissue-specific analyses, yet was based on samples that were collected from recently deceased donors. Consequently, the observed chaperone levels might represent stressful conditions. To test for this, we repeated analyses while excluding known stress-induced chaperones (Fig. 1). We identified similar trends, suggesting that the impact of stress is partial. Repeated analyses by using the HPA transcriptomic dataset that covered 37 tissues resulted in similar trends (Supplementary Figs. 2 and 3). A limitation of these datasets is that transcript levels might not be indicative of protein levels, as the general correlation between them is not high^43,77^. Nonetheless, chaperone transcript levels correlated with their protein levels in matched skeletal muscle samples^43^ (Supplementary Fig. 5A). To further assess the relevance of the transcriptomic profiles, we profiled the proteome of a differentiating mouse myoblast cell line (Fig. 3a). Despite the difference in measured molecules (transcripts versus proteins) and species, we observed a strong agreement between the differential transcriptomic profile of human skeletal muscle and the proteomic profile of differentiated C2C12 mouse myotubes, but not reserve cells (Fig. 3b–d). Agreement was also observed upon comparing the proteomic profile of differentiated C2C12 mouse myotubes to a proteomic profile of human skeletal muscle^43^ (Supplementary Fig. 5). Moreover, human chaperone genes that were at least 2-fold differentially expressed in skeletal muscle by GTEx were also enriched for *C. elegans* muscle chaperones (Fig. 3e), and for chaperones causal for muscle disorders (Fig. 2c), supporting the biological relevance of differential tissue transcriptomic profiles.

A small subset of chaperones, which we denoted as core, did act as consistent building blocks across all tissues. Belonging to fundamental cellular processes (Fig. 4a) this core subset was more highly expressed relative to variable chaperones (Fig. 4d). Core chaperones were also significantly more important for growth than other chaperones (Fig. 4c), and ~2.5-fold less likely to be causal for heritable disorders (9% versus 25%, Fig. 4b), suggesting that they could be lethal when mutated in the germline. Despite their consistent expression, core chaperones had tissue-specific functional relationships both with other core chaperones and with variable chaperones (Fig. 5a, b, Supplementary Fig. 7A).

The existence of ubiquitously expressed chaperones was previously suggested but not quantitatively demonstrated. An aging-related subnetwork of chaperones was defined by the impact of chaperone down-regulation on age-dependent neurodegenerative models^37^. A comparison between our core subset and the aging-related subset revealed an overlap of 10 chaperones (*p* = 8E−5, hypergeometric test, see the “Methods” section) all of which were associated with de novo protein folding or protein maturation. A second study identified an epichaperome, which under stress conditions forms a network of stable complexes that facilitate tumor survival^70^. A comparison between our core subset and this epichaperome revealed an overlap of nine chaperones (*p* = 0.024, see the “Methods” section), six of which were associated with signaling. Taken together, the core set of chaperones that we defined is multifaceted, and its dysregulation has pathological implications in neurodegeneration and cancer.

We tested the generalizability of the tissue-specific chaperone organization across the time axis, focusing on human organ development^42^ and brain aging^37^. Across developing organs and time points, core chaperones were indeed more uniformly expressed than variable chaperones (Fig. 6a). Moreover, functional relationships among chaperones were generally maintained in development (Fig. 6b) and in aging (Fig. 6c). Importantly, focusing on brain aging, we found that brain-required chaperones, including core and brain-upregulated chaperones, were specifically downregulated with age (Fig. 6d), and further decreased in Alzheimer’s disease patients (Fig. 6e). These analyses suggest that the tissue-specific organization of the chaperone network is established in organ development, maintained through adulthood, and is challenged in aging. This organization could therefore contribute to tissue-specific phenotypes and age-dependent diseases.

**Fig. 7:**
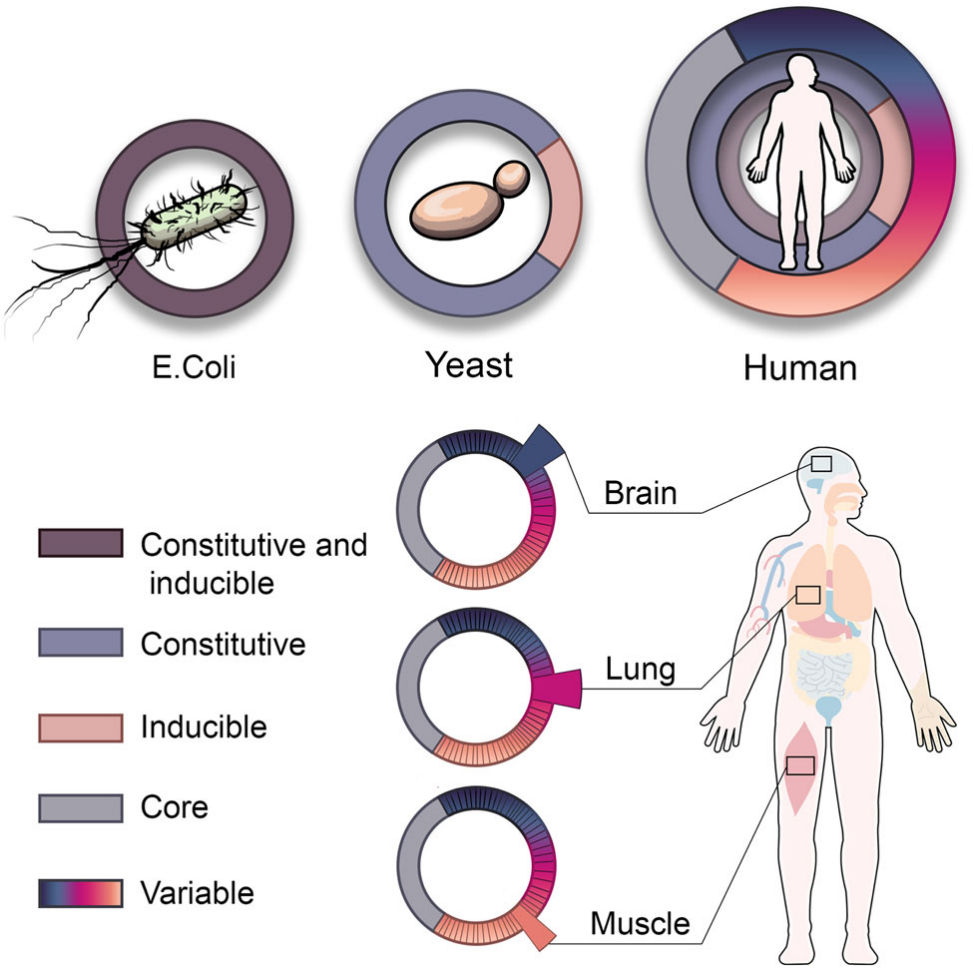
A layered architecture of chaperones across unicellular and multicellular organisms. The chaperone system of *E. coli* is composed of constitutive chaperones that can be induced upon stress (left). The chaperones system in yeast diverged to include chaperones that are either constitutive or stress-induced (middle). In multi-cellular organisms such as human, the chaperone system diverged further to also include core chaperones that are stable across tissues and variable chaperones that are differentially expressed across tissues (right), thereby giving rise to tissue-specific expression patterns, for example in brain, lung, or muscle.

Altogether, our analyses expose a novel layer of functional organization: Core chaperones, involved in basic cellular function shared by all cell-types, behave more like a one size fits all, whereas variable chaperones respond to tissue-specific demands and behave as a tailored system. We thus expand the previous functional view of chaperones, whereby the transition between prokaryotes and eukaryotes separated stress induced from constitutive chaperones^28^. We propose that the transition to multi-cellularity further separated constitutive chaperones into core and variable chaperones, allowing for the modulation of the chaperone system in a tissue-dependent manner (Fig. 7).

The functional organization of chaperones is likely associated with their transcriptional regulation across tissue. In unicellular eukaryotes, chaperones required for de novo folding, such as CCT or Prefoldin subunits and ribosome-associated HSP70, were transcriptionally and functionally linked to the translational machinery^28^, as might still be the case for certain core chaperones. Variable chaperones, in contrast, might be upregulated by tissue-specific transcription factors. In support, neuronal stem and progenitor cell differentiation was shown to rewire the chaperone network^78^. Likewise, the main muscle differentiation transcription factor, MYOD1 (HLH-1 in *C. elegans*), was shown to drive the expression of chaperones in C2C12 mouse myoblast cell line and *C. elegans* muscle^19,79,80^.

Chaperones tissue-tailored organization could offer an explanation for the tissue-specificity of heritable chaperonopathies and protein-misfolding diseases^73,75,81^. These diseases manifest clinically in few tissues, although most genes and chaperones whose aberrations lead to disease are expressed ubiquitously (Fig. 2a)^34,53,55^. In some cases, this could be due to the overexpression of such genes in their disease-manifesting tissue^34,55,56,82^, as we observed for muscle diseases (Fig. 2c). We propose that it could also stem from chaperones collaborating or competing with each other over substrates, thereby leading to a proteostatic environment that is limiting to other proteins^83–87^. Since the levels of chaperones and substrates are altered between tissues, and since these alterations are functional, the proteostatic environment could both contribute to tissue-specific functionality and elicit tissue-specific phenotypes when perturbed, thereby giving rise to tissue-specific protein misfolding diseases. Targeting ubiquitously expressed chaperones can give rise to non-specific effects and has therapeutic limits^88^. Our results can help direct efforts toward chaperones that are more sensitive to cell-specific modulation of their expression, and thus might be better suited as potential therapeutic targets for cell-specific diseases. Formulating and quantifying the function of the chaperone system at large scale and within single cells is therefore one of the main challenges in proteostasis research.

## Methods

### Curation of an integrated list of human chaperones

We compiled a curated list of 195 known human chaperone, co-chaperone, and folding enzyme genes, collectively named chaperones (Supplementary Data 1). For that, we compared two curated lists of human chaperones: (1) a list of 332 chaperones curated by considering their biochemical properties and protein domains^37^; and (2) a list of 194 chaperones complied based on sequence homology to conserved canonical chaperones and supplemented by a list of known co-HSP90 chaperones^6,89^. These lists included 114 genes belonging to well-conserved chaperone families, including sHsp (10 genes), HSP60/HSP10 (15 genes), HSP70 (13 genes), HSP90 (4 genes), Prefoldin (9 genes), and co-chaperones Hsp40 (49 genes) and NEFs (14 genes). 106 of these chaperones appeared in both lists (~93% overlap). Less agreement between the lists was observed for the co-Hsp90 family, where only 24 (19%) chaperones out of 127 putative co-HSP90 chaperones appeared in both lists. This inconsistency was due to the lack of a conserved domain shared by all members of the co-HSP90 family, and uniquely by them. For example, many known co-HSP90 chaperones have a tetratricopeptide repeat (TPR) motif, but not all TPR-containing proteins are chaperones^90^. We therefore included 34 co-HSP90 chaperones that were previously shown to interact or function with HSP90^15,91,92^, three of which did not appear in any list and 22 appeared in both lists (~71% overlap). The remaining genes in the initial lists^6,37,89^, denoted as ‘others’, included members such as ER chaperones, mitochondrial chaperones, AAA+ proteins and folding enzymes. For each member, we mined the literature to determine whether it functions as a chaperone that transiently assists the folding, unfolding, assembly or disassembly of proteins and protein complexes. For example, we excluded eight nuclear cyclophilins that appeared in both lists, as these were recently shown to constitute an integral part of spliceosomal complexes and were renamed as spliceophilin^93^. Of the 47 chaperones that we included as ‘others’, 30 appeared in both lists (~64% overlap). Finally, we excluded genes that were annotated as pseudogenes or as noncatalytic proteins. For example, of the six HSP90 family genes, we excluded two pseudogenes (HSP90AA2 and HSP90B2P). Out of the 195 chaperones, only PPIAL4A did not meet the expression threshold of 1 TPM in any tissue and was therefore omitted from further analyses.

### Additional chaperone subsets

We annotated the functionality of core chaperones by manual curation of their Uniprot entries^94^ and by literature search. Data of curated, muscle chaperones in *C. elegans* were obtained from ref. ^19^. Data of orthologous chaperones in *C. elegans* were obtained via the OrthoList2 tool for comparative genomic analysis between *C. elegans* and humans^95^, and each human chaperone was fitted with the *C. elegans* gene(s) that was identified as its orthologue by the maximal number of programs. Data of orthologous chaperones in mouse were obtained from Mouse Genome Informatics (MGI) website^96^.

Genes with a known genetic aberration that is causal for heritable diseases were obtained from OMIM^54^, and limited to genes with a known genetic basis regardless of their mode of inheritance. Manually curated data of the tissues that clinically manifest a Mendelian disease were obtained from ref. ^53^. We additionally mined the literature for chaperones that are causal for or associated with muscle disorders or muscle function (Supplementary Data 3 and Supplementary Table 1).

Chaperones that are induced by stress were obtained from a meta-analysis study of several microarray datasets of stress-induced genes^89^.

#### Comparison to additional functional subsets

An aging-related subnetwork of chaperones was defined previously by the impact of chaperone down-regulation on age-dependent neurodegenerative models^37^. The study included 187 chaperones, of which 20 were core chaperones. 28/187 were considered as an aging-related subset, including 10 core chaperones (*p* = 8E−5, hypergeometric test). The 10 core chaperones included 6 CCT subunits, HSPA14, DNAJA1, HSP90AA1 and its cochaperone STIP1. A cancer-related study identified an epichaperome, which under stress conditions forms a network of stable complexes that facilitate tumor survival^70^. The study included 63 chaperones, of which 13 were core chaperones. 26/63 chaperones formed the epichaperome, including 9 core chaperones (*p* = 0.024, hypergeometric test). These included HSP90AA1 and its co-chaperones (DNAJC7, AHSA1, NUDC, SGTA, STIP1, SUGT1), as well PFDN2, DNAJA1. The core chaperones DNAJA1, HSP90AA1, and its cochaperone STIP1, were part of both the aging-related and the epichaperome subsets.

### Human gene expression dataset

Data of transcriptomic profiles of human tissues measured via RNA-sequencing were downloaded from the GTEx portal (version 7). We analyzed samples from donors with traumatic injury fitting with sudden death, and avoided other death causes to limit the effect of treatment and stress on chaperone expression^44–47^. We considered only physiological tissues (i.e., no transformed cells) with ≥5 samples. Brain sub-tissues were collapsed into three main regions including brain basal ganglia, largely cortex, and brain other, according to Paulson et al. ^97^. Altogether, we analyzed 488 transcriptomic profiles and 29 tissues (Supplementary Data 2). Genes were mapped to their Ensembl gene identifiers using BioMart^98^, and filtered to include only protein-coding genes. Data of gene expression pattern during organ development was extracted from ref. ^42^, and included organ and timepoint specificity scores per gene, and module association per gene and organ. Data of chaperone expression in aging brain were extracted from ref. ^37^, and included, per chaperone, a correlation between its expression and age, a ratio between its expression in patients with a neurodegenerative disease and age-matched controls, its module association, and a list of highly correlated chaperone pairs. To verify the trends observed with the GTEx dataset, we used transcriptomic profiles of 37 human tissues measured via RNA-sequencing by the HPA^30^. Expression values in protein TPM (pTPMs) units were downloaded from the HPA portal.

### Gene expression analyses

To analyze the expression pattern of genes across tissues, we considered genes as expressed in a tissue if their median expression level was ≥1 TPM. Results pertaining to expression thresholds of 5 or 10 TPM are presented in Supplementary Fig. 1 and Supplementary Fig. 3. The differential expression of genes per tissue was calculated as in ref. ^57^. Raw reads were normalized to obtain the same library size for every sample by using the trimmed mean of *M*-values (TMM) method by the edgeR package^99^. Genes with ≤10 raw counts per sample across all samples were removed before normalization. In each sample, we transformed the normalized counts profile using the voom method^100^. To compute the differential expression of genes in a given tissue relative to their expression in other tissues, we compared all transformed profiles of that tissue to a background set containing transformed profiles of all other tissues. Differential expression of genes in a tissue relative to other tissues was then calculated by using the Limma linear model^101^. The differential expression of a gene in a given tissue was then set to its log_2_ fold-change value in that tissue, and the *p*-value was adjusted for multiple hypothesis testing via Benjamini–Hochberg correction. We considered genes with an absolute log_2_ fold-change ≥1 and adjusted *p*-value ≤ 0.05 as differentially or variably expressed. We defined core chaperones as chaperones that across all tissues had less than a 2-fold change in their expression. In the comparison between organ development and adult tissues, cerebrum and cerebellum were compared to brain 1 and brain 2, respectively. In the analyses of chaperone expression in different regions of the aging brain and in patients with neurodegenerative diseases versus controls, brain regions were matched with brain 1 for calling upregulated and downregulated variable genes.

### Impact on growth analyses

The impact on growth score of a gene was measured by the change in growth rate of a cell line containing a CRISPR-induced inactivation of the gene relative to control^69^, denoted as CRISPR score of the gene. A negative (or positive) gene value implied a reduced (or increased) growth rate of its respective CRISPR-inactivated cell line relative to control; genes with negative values were considered important for growth of the respective cell lines. Data of CRISPR scores were obtained from DepMap^41^ and from Project Score^40^, via the Broad Institute’s DepMap portal (https://depmap.org). DepMap data were available for 186 chaperones across 769 cell lines. Project Score data were available for 185 chaperones across 318 cell lines. Analyses were performed using data from DepMap. We repeated them using data from Project Score, which led to similar results (Supplementary Fig. 4).

### Statistical analyses

To test the null hypothesis that the expression levels across tissues of chaperones and protein-coding genes were drawn from the same distribution we used the KS test. We used the same test to compare between the impact of growth values of chaperones and protein-coding genes. To test the null hypothesis that gene expression levels of chaperones and protein-coding genes are similar, we used the MW *U* test. To test the null hypothesis that the impact of growth values of chaperones were similar to those of other protein-coding genes, we considered per gene its minimum CRISPR score across cell lines per project, and applied the KS test. To compute the statistical significance of the overlap between different gene sets we used Fisher exact test. Upon analyzing orthologous chaperones and the significance of the overlap, we considered all human chaperones in our data that had an orthologue in the respective species. To assess the correlation between the differential expression of human genes and mouse proteins in muscle samples we computed their Pearson correlation. To test the null hypothesis that organ and time point specificities were similar between chaperones and protein-coding genes, or between core and variable chaperones, we used the MW *U* test. The same test was used to check the null hypothesis that chaperone pairs have similar correlated expression in adult tissues, whether they belong to the same developmental module or not. *p*-values were adjusted for multiple hypothesis testing by using Benjamini–Hochberg procedure.

### Coexpression analyses

For each pair of chaperones and each tissue, we calculated the Spearman correlation between the expression levels of the two chaperones across all samples of that tissue. These correlations were viewed as a heatmap (Fig. 5 and Supplementary Fig. 7).

### ChaperoneNet implementation

The ChaperoneNet server was implemented in Python by using the Flask framework with data stored on a MySQL database. The website client was developed using the ReactJS framework and designed with Semantic-UI. The network view is displayed by the cytoscape.js plugin^102^. The website supports all major browsers. Recommended viewing resolution is 1440 × 900 and above.

### Cell cultivation of C2C12 mouse myoblast cell line and RT-qPCR analysis

C2C12 cells (ATCC CRL1772) were grown in Dulbecco’s modified Eagle medium (DMEM) containing 10% fetal bovine serum (FBS), penicillin/streptomycin (0.1 mg/mL) and l-glutamine (2 mM). For differentiation, cells were grown to 95% confluency and medium was changed to differentiation medium, containing 4% donor horse serum (DHS) instead of FBS. During differentiation, the medium was changed at least every other day. Cells were harvested after 8 days, when they reached maximal differentiation. To separate myotubes from reserve cells, we used the property of myotubes to detach from cell culture plates under milder conditions than reserve cells. Plates were washed with PBS and incubated with trypsin diluted in DMEM medium without additives until most myotubes started detaching. Myotubes were then washed off the plate using PBS buffer. The remaining reserve cells were detached using 0.25% Trypsin and 0.05% EDTA solution. Both myotubes and reserve cell fractions were washed twice with 10 mL PBS and kept at −80 °C. To verify that C2C12 myotubes did differentiate, the expression of several gene markers, including myosins, MYH3, MYOM1, and chaperones, HSPB7 and UNC45B, was determined. Total RNA was purified using GENEzol TriRNA Pure Kit (Geneaid) and DNase I DNA-free DNA removal Kit (Invitrogen). First-strand cDNA was generated using Iscript cDNA Synthesis Kit (Biorad) for real-time PCR (qPCR). For each reaction 1 µg of total RNA was used. The cDNA was stored at −20 °C or used immediately. cDNA was amplified in CFX96 C1000 Thermal cycler (Bio-Rad) using KAPA SYBR FAST Kit (Sigma). For each reaction 20 ng of cDNA was used. The real-time PCR was performed as follows: one cycle of denaturation (95 °C for 3 min) followed by 40 cycles of amplification (95 °C for 3 s, 60 °C for 30 s). Each reaction was monitored by the use of a negative control (no template). DNA amounts were quantified using the ΔΔCt method, normalizing to the housekeeping gene Hprt1. MW test was used for comparisons between myotubes or reserve cells to control (Supplementary Fig. 5E). A list of primers used is detailed in Supplementary Table 2.

### C2C12 mouse myoblast cell line mass-spectrometric analysis

The proteome of C2C12 mouse myoblast cell line before differentiation (day 0); after 8 days of differentiation to myotubes (day 8, myotubes); and of undifferentiated muscle cells (day 8 reserve cells) were analyzed using LC–MS/MS (*n* = 3 biological repeats for each treatment; Technion, Israel). Cells were suspended with 9 M urea and 10 mM DTT solution and sonicated with a Cup-Horn sonicator for 2 min with 10 s on and off cycles. After centrifugation to remove cell debris, samples were digested by trypsin (Promega) at a 1:50 enzyme-to-substrate ratio, overnight at 37 °C with an additional trypsinization done for 4 h. The tryptic peptides were desalted using C18 tips (Top tip, Glygen) dried and re-suspended in 0.1% formic acid. The resulted peptides were analyzed by LC–MS/MS using a Q-Exactive plus mass spectrometer (Thermo) fitted with a capillary HPLC (easy nLC 1000, Thermo-Fisher). The peptides were loaded onto a C18 trap column (0.3 × 5 mm, LC-Packings) connected on-line to a homemade capillary column (20 cm, 75 micron ID) packed with Reprosil C18-Aqua (Dr. Maisch GmbH, Germany) in solvent A (0.1% formic acid in water). The peptides mixture was resolved with a (5–28%) linear gradient of solvent B (95% acetonitrile with 0.1% formic acid) for 180 min followed by gradient of 15 min gradient of 28–95% and 25 min at 95% acetonitrile with 0.1% formic acid in water at flow rates of 0.15 μL/min. Mass spectrometry was performed in a positive ion mode (at mass range of *m*/*z* 350–1800 AMU and resolution 70,000) using repetitively full MS scan followed by collision induces dissociation (HCD, at 35 normalized collision energy) of the 10 most dominant ions (>1 charges) selected from the first full MS scan.

The mass spectrometry data was analyzed using the MaxQuant software 1.5.2.8. (www.maxquant.org) for peak picking identification and quantitation using the Andromeda search engine, searching against Mus musculus part of the Uniprot database (June 2015) with mass tolerance of 20 ppm for the precursor masses and 20 ppm for the fragment ions. Trypsin was set as the protease. Methionine oxidation and acetylation of protein N-term were accepted as variable modifications and carbamidomethyl on cysteine was accepted as static modification. Minimal peptide length was set to six amino acids and a maximum of two miscleavages was allowed. Peptide-level and protein-level false discovery rates (FDRs) were filtered to 1% using the target-decoy strategy. The protein table was filtered to eliminate the identifications from the reverse database, and common contaminants. The data was quantified by label free analysis using the MaxQuant software, based on extracted ion currents (XICs) of peptides enabling quantitation from each LC/MS run for each peptide identified in any of experiments. Welch’s *t*-test was performed on the log_2_ of the LFQ Intensity values using Perseus software. We used only mouse proteins whose expression was measured at day 0 and day 8 and that showed significant differences, and their human homologs. In the comparison to protein abundance in human skeletal muscle^43^, human proteins were associated with their median raw protein abundance across skeletal muscle samples.

### Cell cultivation of LHCN-M2 human myoblast cell line and RT-qPCR analyses

LHCN-M2 (cycling human myoblasts) cells were described elsewhere^103^. The cells were maintained in Ham-F12 supplemented with 100 U/mL penicillin/streptomycin, 20% FBS, and 25 ng/mL of rhFGF-b/FGF-2. For induction of myogenic differentiation, LHCN-M2 cells were cultured in DMEM supplemented with 100 U/mL penicillin/streptomycin, and 30 μg/mL human insulin solution for 5 days. Cells were routinely tested for mycoplasma contamination.

For gene expression analysis in LHCN-M2 cells, total RNA was isolated using Trizol, followed by purification using RNA Clean & Concentrator (Zymo Research, USA) and DNase I Set (Zymo Research, USA). First-strand cDNA was generated using Maxima First Strand cDNA Synthesis Kit for RT-qPCR (ThermoFisher). For each reaction 1 µg of total RNA was used. The samples were incubated at 25 °C for 10 min followed by a step of 50 °C for 15 min, and then 85 °C for 5 min. The cDNA was stored at −20 °C or used immediately for RT-qPCR. cDNA was amplified in CFX96 Touch Thermal cycler (Bio-Rad) using TAQ SYBR Green qPCR SYBR (ThermoFisher). For each reaction 50 ng of cDNA was used. RT-qPCR was performed as follows: one cycle of denaturation (95 °C for 3 min) followed by 40 cycles of amplification (95 °C for 10 s, 60 °C for 30 s). Each reaction was monitored by the use of a negative control (no template). DNA amounts were quantified using the ΔΔCt method, normalizing to RPL0and the cycling condition was set to 1. A list of primers used is detailed in Supplementary Table 2. Student’s *t*-test was used for comparisons between two groups.

### Reporting summary

Further information on research design is available in the Nature Research Reporting Summary linked to this article.

## Supplementary information

Supplementary Information

Description of Additional Supplementary Files

Supplementary Data 1

Supplementary Data 2

Supplementary Data 3

Supplementary Data 4

Supplementary Data 5

Supplementary Data 6

Reporting Summary

## Data Availability

Data of chaperones analyzed in the study are available as Supplementary Data 1. Data of tissues analyzed in the study appear in Supplementary Data 2. Data of disease-associated chaperones appear in Supplementary Data 3. Data of expression fold-change of chaperones across tissues is available as Supplementary Data 4. Data of pairwise chaperone co-expression correlations per tissue are available as Supplementary Data 5, and through the ChaperoneNet webtool (https://netbio.bgu.ac.il/chapnet/). The mass spectrometry proteomics data were deposited in ProteomeXchange Consortium via the PRIDE^104^ partner repository with the dataset identifier PXD022678 (LC–MS/MS of C2C12 mouse myoblast cell line). The analyzed mass spectrometry proteomics data of mouse myoblasts are available as Supplementary Data 6. Source data for Figs. 1–6 are provided with this paper. The following databases were used in the study: GTEx v7 http://www.gtexportal.org/home/; The Human Protein Atlas http://www.proteinatlas.org; Mouse Genome Informatics http://www.informatics.jax.org/; BioMart http://m.ensembl.org/biomart/martview; OMIM http://www.omim.org/; and DepMap http://depmap.org.

## Source data

Source Data
