# Supplementary material for: The landscape of molecular chaperones across human tissues reveals a layered architecture of core and variable chaperones
Source: Nat Commun. 2021 Apr 12;12:2180. doi: 10.1038/s41467-021-22369-9 (PMC8042005; doi:10.1038/s41467-021-22369-9)
Supplement: Supplementary file 1 — Supplementary Information [file 41467_2021_22369_MOESM1_ESM.pdf]

Supplementary Information

The landscape of molecular chaperones across human tissues reveals a layered architecture of core-variable chaperones

Shemesh, Jubran, et al.

Supplementary Tables 1,2

Supplementary Figures 1-8

Supplementary References

Supplementary Table 1: Chaperones that were expressed at least 2-fold higher in human skeletal muscle relative to other tissues and their association with muscle disease or function.

Chaperone name	Chaperone ENSG ID	Fold Change value (Log_2FC)	Upregulated in *C. elegans* muscle	Muscle association (reference)	
				Disease*	Function
UNC45B	ENSG00000141161	9.46	Yes		[1,2]
HSPB3	ENSG00000169271	6.07	Yes	Yes	[3,4]
HSPB7	ENSG00000173641	5.31		Yes[5,6]	[5-8]
HSPB6	ENSG00000004776	4.88	Yes	[9]	[10]
CRYAB	ENSG00000109846	3.62	Yes	Yes	[11]
HSPB8	ENSG00000152137	3.55	Yes	[12-14]	[15,16]
BAG3	ENSG00000151929	3.13		Yes	[16,17]
HSPB2	ENSG00000170276	3.13	Yes		[3,4,18]
FKBP3	ENSG00000100442	3.04			
DNAJB5	ENSG00000137094	2.88	Yes	[19]	[19]
FKBP5	ENSG00000096060	2.86			[20,21]
HSPA2	ENSG00000126803	2.84	Yes	[22,23]	[24]
DNAJA4	ENSG00000140403	2.61	Yes		[25]
DNAJB6	ENSG00000105993	2.24	Yes	Yes	[15,16,26]
CDC37L1	ENSG00000106993	2.00		[27]	
DNAJC5B	ENSG00000147570	1.98	Yes	[28]	
SACS	ENSG00000151835	1.96			
DNAJA3	ENSG00000103423	1.95	Yes	[29]	[30,31]
DNAJC28	ENSG00000177692	1.90			
VBP1	ENSG00000155959	1.84			
PPID	ENSG00000171497	1.83		[32]	[32]
TRAP1	ENSG00000126602	1.82		[33]	
HSPA9	ENSG00000113013	1.81		[34,35]	[36]
HSPA8	ENSG00000109971	1.77	Yes	[37]	[15,16,38]
DNAJC19	ENSG00000205981	1.75		Yes [39,40]	[40]
CLPX	ENSG00000166855	1.67			
DNAJC21	ENSG00000168724	1.64			
VCP	ENSG00000165280	1.58		Yes	[41,42]
DNAJB4	ENSG00000162616	1.52	Yes		[43]
TOMM70A	ENSG00000154174	1.50		[44]	
HSPA1L	ENSG00000204390	1.50	Yes		
HSPB1	ENSG00000106211	1.48	Yes		[45]
RPAP3	ENSG00000005175	1.42			
DNAJC12	ENSG00000108176	1.37			
HSPA4	ENSG00000170606	1.37	Yes	[46]	[46]
TXNRD1	ENSG00000198431	1.36			[47]
DNAJC11	ENSG00000007923	1.33			
GRPEL1	ENSG00000109519	1.32			
HSP90AB1	ENSG00000096384	1.31	Yes	[35,48]	[49]
PHB	ENSG00000167085	1.29			[50,51]
BAG1	ENSG00000107262	1.27		[52-54]	[15,16]
PHB2	ENSG00000215021	1.25			[55-57]
CCT6B	ENSG00000132141	1.25	Yes		[58]
BAG6	ENSG00000204463	1.20			
DNAJC24	ENSG00000170946	1.17			
DNAJB7	ENSG00000172404	1.17	Yes		
DNAJC16	ENSG00000116138	1.16	Yes		
CDC37	ENSG00000105401	1.13			[59-61]

TTC4	ENSG00000243725	1.12			
PPIC	ENSG00000168938	1.11			
CCT7	ENSG00000135624	1.10	Yes		[58]
CCT8L2	ENSG00000198445	1.10	Yes		[58]
STUB1	ENSG00000103266	1.08	Yes	[62,63]	[64,65]
ST13	ENSG00000100380	1.06	Yes		
CLPB	ENSG00000162129	1.06			
DNAJA2	ENSG00000069345	1.02	Yes		[25]
BAG2	ENSG00000112208	1.01	Yes	[66]	[67]

* The disease associations for chaperones denoted with 'Yes' appear in Supplementary data 3.

Supplementary Table 2: A list of primers used in this study.

Gene target	Organism	Forward primer	Reverse primer
HPRT1	Mouse	ATGGACTGATTATGGACAGGACTG	TCCAGCAGGTCAGCAAAGAAC
MYH3	Mouse	TCTCTGTCACAGTCAGAGGTGT	AAAGGTGCAGCTATGCCAAAC
MYOM1	Mouse	GAATGCAGCTGGACTTAGCG	GAGACACTCCTCCCCCGATA
HSPB7	Mouse	ACCCAGATAGACCCTCCCTAT	GTTAGACTCTGCCCTCGTCT
UNC45B	Mouse	CTCCAGGGTACAGACGATGT	GGACAATGAGGTTGTTGGCA
RPL0	Human	TTAAACCCTGCGTGGCAATCC	CCACATTCCCCCGGATATGA
MYOG	Human	CACTCCCTCACCTCCATCGT	CATCTGGGAAGGCCACAGA
HSPB1	Human	TCCAACGAGATCACCATCC	CTAAGGCTTTACTTGGCGG
HSPB2	Human	CATGGTCCACAATGTATGGT	ATTTGGGTTTATTCAGCTCCAC
HSPB3	Human	GACTAAGTGACATCGTATCGG	ACAAACATTCTCGTAGTACCAG
HSPB4	Human	CTTAGGGTCTCAGGGTGTC	GGCTGCTATCTAAAGGAGTG
HSPB5	Human	CCCAAGAAATAGATGCCCT	GTCACAAGACTTTCATTCACTG
HSPB6	Human	CTACCAGCACTACCCTAACC	TCAGAAGGAAGTAGAGGAGG
HSPB7	Human	CTGTGCTTTGTCACTGAAGG	GAAATACTCCTCAGTGCCCA
HSPB8	Human	TTTCCACATTAGCACTCCC	AAATCCCGACTTAAACACAG
HSPB9	Human	CCAATGGCTGATGGTGAC	CACCTTCTGTGACATGCG
HSPB10	Human	ATTTCCCAAAGGTACTCACAG	TCTGTCCACCTTCTTTATGTC

Supplementary Figures

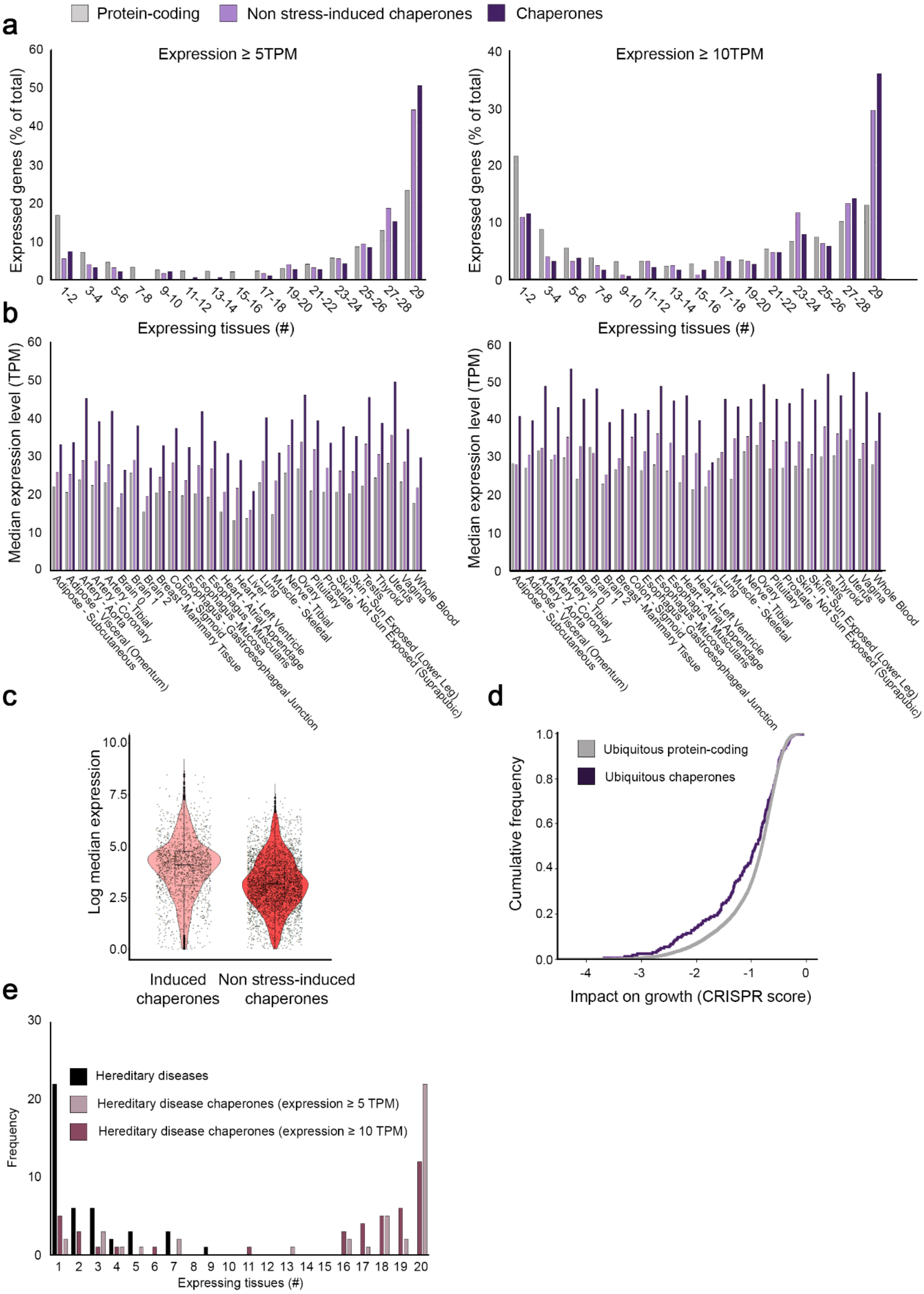

Supplementary Figure 1. Chaperones are highly and ubiquitously expressed across tissues when tested at additional expression thresholds.

a. The distribution of chaperones, non stress-induced chaperones and other protein-coding genes by the number of tissues expressing them at a level ≥ 5 TPM (left, 192 chaperones, 129 non stress-induced chaperones and 16,390 protein-coding genes) or ≥ 10 TPM (right, 191 chaperones, 128 non stress-induced chaperones and 15,178 protein-coding genes). For 5 TPM, chaperones and non stress-induced chaperones were significantly more ubiquitously expressed than other protein-coding genes (p=0.002 for both, two-sided Kolmogorov-Smirnov (KS) test). A somewhat weaker trend was observed for 10 TPM (p=0.06 and p=0.1, respectively, two-sided KS test).

b. The median expression levels per tissue of chaperones, non stress-induced chaperones and other protein-coding genes. Only genes expressed at a level ≥ 5 TPM (left) or ≥ 10 TPM (right) were considered. Chaperones tend to be significantly more highly-expressed across all 29 tissues (adjusted p ranged from 0.000137 to 4.6E-12 for 5 TPM, and from 0.016 to 1.03E-7 for 10 TPM, two-sided Mann-Whitney (MW) test). Non stress-induced were significantly more highly expressed in 27/29 tissues for 5 TPM (adjusted p ranged from 0.0017 to 0.028) and in 8/29 tissues for 10 TPM (adjusted p=0.042). For 5 TPM chaperones n=122 to 179; non stress-induced chaperones n=45 to 119; other protein-coding genes n=6,643 to 11,886. For 10 TPM n=96 to 166; non stress-induced chaperones n=45 to 108; other protein-coding genes n=4,254 to 9,614.

c. The median expression levels per tissue of stress-induced (n=1,097) versus non stress-induced chaperones (n=4,003). Stress-induced chaperones were significantly more highly expressed (p=2.2E-16, one-sided MW test). In the boxplot representation, center line, median; box limits, upper and lower quartiles; whiskers, 1.5x interquartile range.

d. The cumulative distribution of the impact on growth of the subsets of 165 chaperones and 9,714 other protein-coding genes that were expressed ubiquitously (i.e., in all tissues). Impact on growth was measured in 769 cell lines harboring CRISPR-induced gene inactivation (CRISPR score) by the DepMap project. Each gene was associated with its minimal CRISPR score. Chaperones tend to be more important for growth than other ubiquitously expressed genes (p=0.0025, one-sided KS test).

e. Manifestation of hereditary diseases that are caused by aberrant chaperones, and the tissue distribution of these chaperones. Most diseases manifest clinically in a tissue-specific manner (heredity disease). In contrast, the chaperones whose aberration leads to these diseases are expressed ubiquitously across tissues, upon considering expression thresholds of 5 TPM or 10 TPM (heredity disease chaperones). Sub-parts of the same tissue were united (e.g., adipose subcutaneous and adipose visceral omentum were united into a single adipose tissue), resulting in 20 tissues.

Source data are provided as a Source Data file.

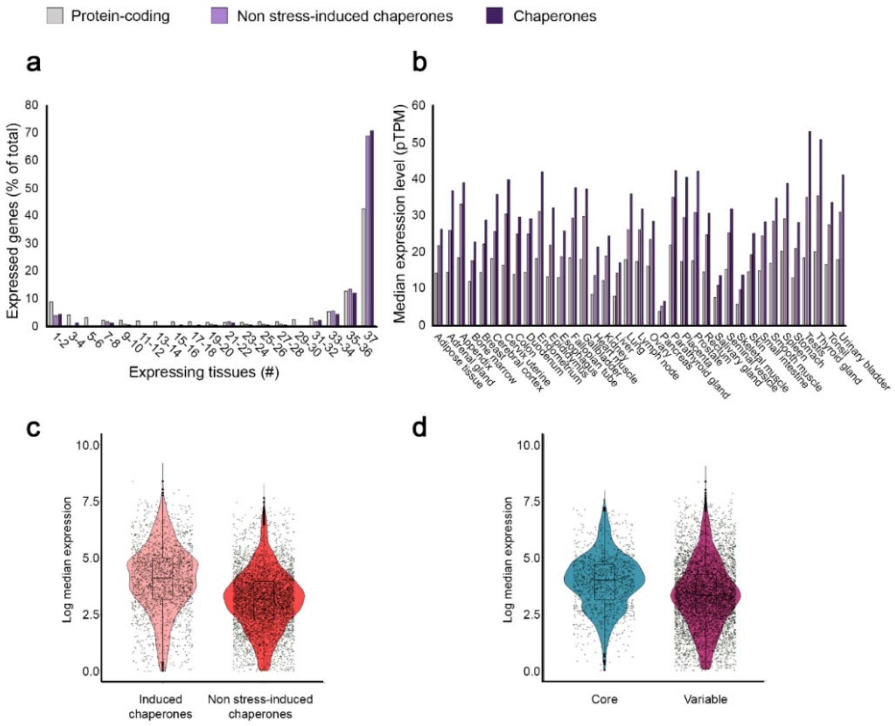

Supplementary Figure 2. Chaperones are broadly expressed across 37 tissues profiled by the Human Protein Atlas.

a. The distribution of 191 chaperones, 128 non stress-induced chaperones and 18,388 other protein-coding genes by the number of tissues expressing them at a level ≥1 pTPM (the first bin represents genes expressed in a single tissue or two tissues, the second bin represents genes expressed in three or four tissues, etc.). Chaperones and non stress-induced chaperones were significantly more broadly expressed than other protein-coding genes (*p*=1E-13 and *p*= 3.5E-06, respectively, two-sided KS test).

b. The median expression levels per tissue of chaperones, non stress-induced chaperones and other protein-coding genes. Only genes expressed at a level ≥1 pTPM were considered. Chaperones and non stress-induced chaperones tend to be significantly more highly expressed than other protein-coding genes across all 37 tissues. Adjusted *p* for chaperones ranged from 4.6E-7 to 6.8E-18; for non stress-induced chaperones from 0.017 to 1.4E-6, two-sided MW test. Chaperones n= 146 to 185; non stress-induced chaperones n= 94 to 123; other protein-coding genes n=9,957 to 16,200.

c. The median expression levels per tissue of stress-induced (n=2,214) versus non stress-induced (n=4,573) chaperones. Stress-induced chaperones were significantly more highly expressed (*p*=2.2E-16, one-sided MW test).

d. The median expression levels of core (n=1,147) versus variable (n=5,641) chaperones. Core chaperones were significantly more highly expressed (*p*=2.2E-16, one-sided MW test).

In the boxplot representation, center line, median; box limits, upper and lower quartiles; whiskers, 1.5x interquartile range.

Source data are provided as a Source Data file.

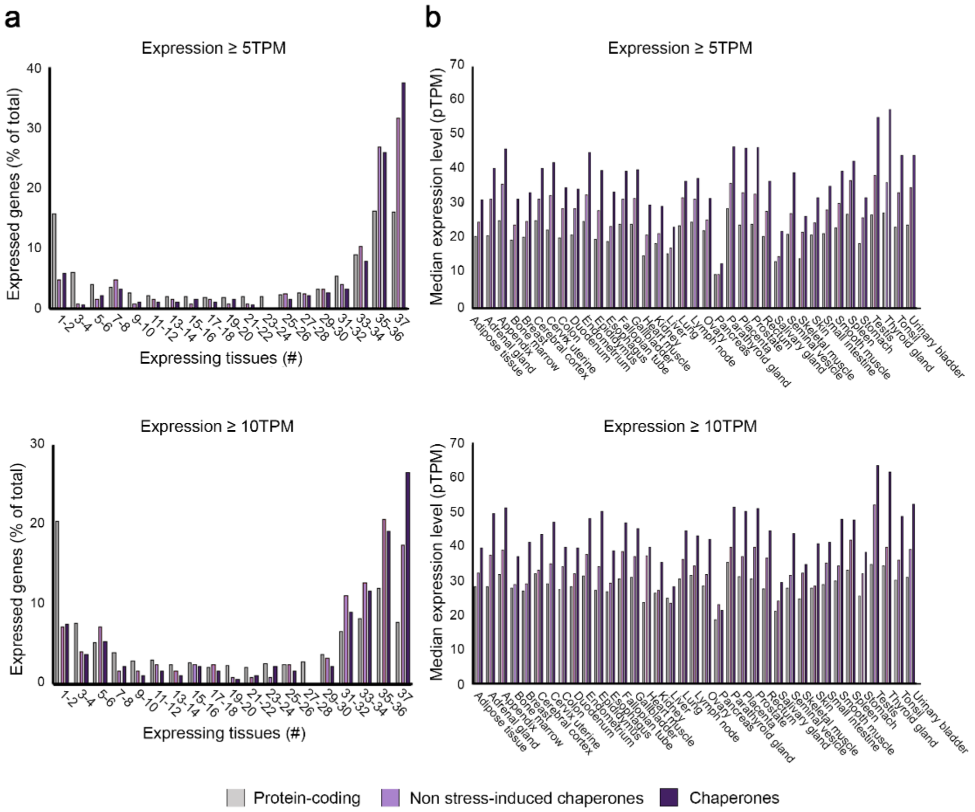

Supplementary Figure 3. Chaperones are broadly expressed across 37 tissues profiled by the Human Protein Atlas when tested at additional expression thresholds.

a. The distribution of chaperones, non stress-induced chaperones and other protein-coding genes by the number of tissues expressing them at a level ≥ 5 pTPM (top, 190 chaperones, 127 non stress-induced chaperones and 17,300 protein-coding genes) or ≥ 10 pTPM (bottom, 188 chaperones, 126 non stress-induced chaperones and 16,274 protein-coding genes). Chaperones and non stress-induced chaperones were significantly more ubiquitously expressed than other protein-coding genes (5pTPM: p = 3.5E-06 and p = 1.3E-07, respectively; 10pTPM: p = 7E-07 and p =1.5E-5, respectively, two-sided KS test).

b. The median expression levels per tissue of chaperones, non stress-induced chaperones and other protein-coding genes. Only genes expressed at a level ≥5 pTPM (top) or ≥10 pTPM (bottom) were considered. Chaperones tend to be significantly more highly-expressed than protein-coding genes across all 37 tissues at the 5 pTPM threshold, and across 35 tissues (excluding liver and pancreas) at the 10 pTPM threshold (5 pTPM: adjusted p ranged from 0.0034 to 3.6E-11; 10 pTPM: adjusted p ranged from 0.002 to 1.93E-8, two-sided MW test). This trend was observed for non stress-induced chaperones relative to protein-coding genes (5 pTPM: 32/37 tissues, adjusted p ranged from 0.044 to 0.0044, two-sided MW test). For 5 TPM chaperones n=91 to 173; non stress-induced chaperones n=52 to 114; other protein-coding genes n=4,064 to 12,793. For 10 TPM n=58 to 162; non stress-induced chaperones n=26 to 104; other protein-coding genes n=1,979 to 10,595.

Source data are provided as a Source Data file.

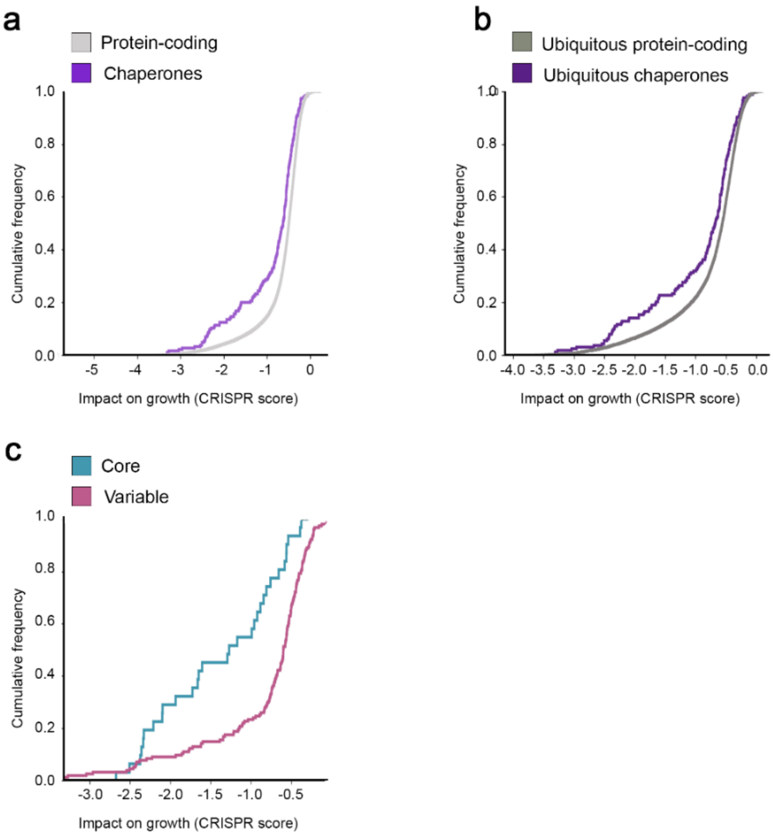

Supplementary Figure 4. The impact on growth of chaperones and other protein-coding genes as measured in 318 cell lines by Project Score. Each gene was associated with its minimal CRISPR score**.**

a. The cumulative distribution of the impact on growth of 185 chaperones and 16,727 other protein-coding genes. Chaperones were significantly more important for growth than other protein-coding genes (p=8.4E-9, one-sided KS test).

b. The cumulative distribution of the impact on growth of the subsets of 163 chaperones and 9,688 other protein-coding genes that were expressed ubiquitously in all tissues. Chaperones tend to be more important for growth even when compared to other ubiquitously expressed genes (p=2E-4, one-sided KS test).

c. The cumulative distribution of the impact on growth of core versus variable chaperones. 31 core and 154 variable chaperones for which CRISPR scores were available were considered. Core chaperones were significantly more important for growth (p=1.02E-5, one-sided KS test).

Source data are provided as a Source Data file.

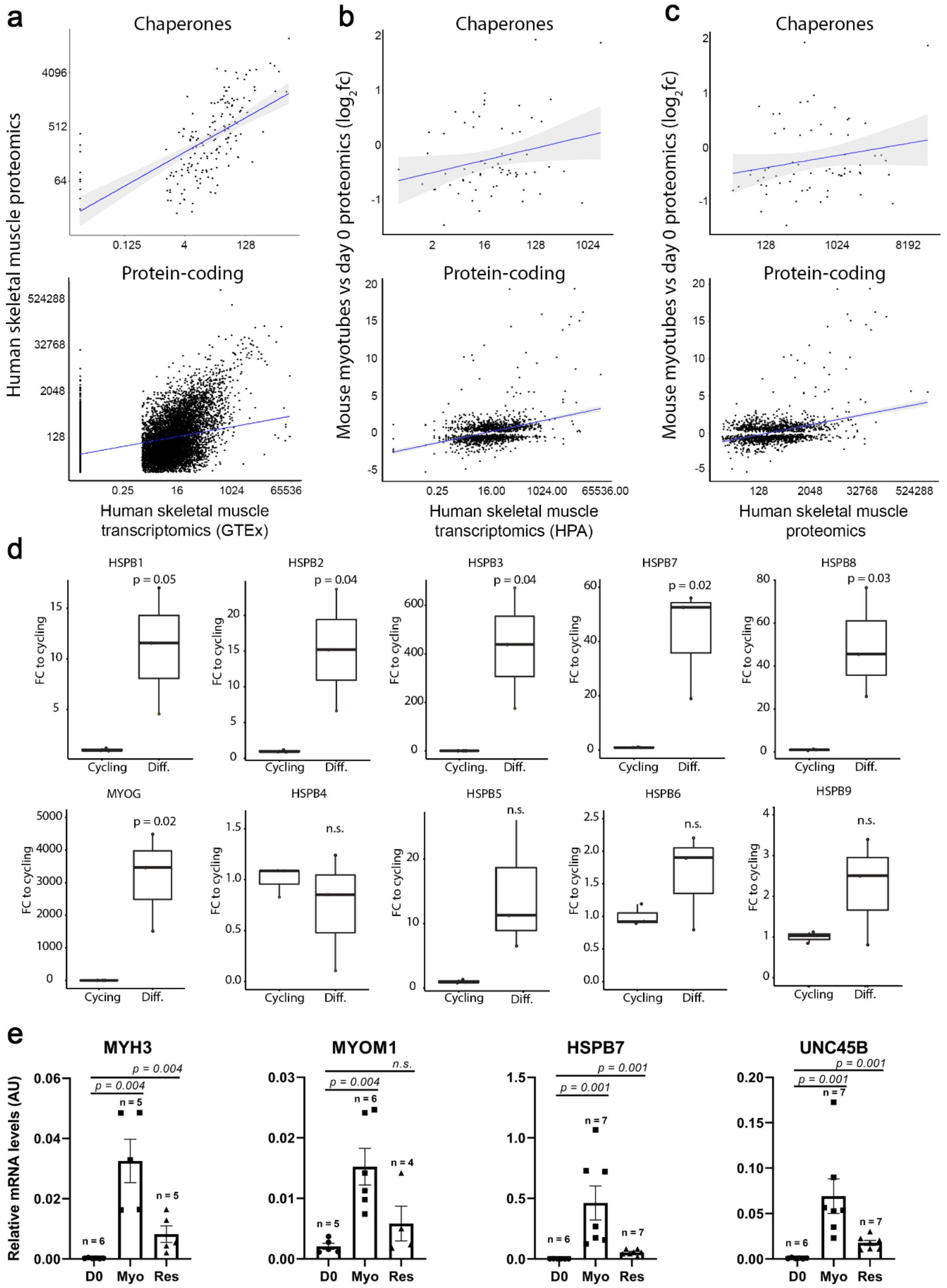
a
b
c
d
e
Chaperones
Protein-coding
Human skeletal muscle proteomics
Mouse myotubes vs day 0 proteomics (log2 fc)
Human skeletal muscle transcriptomics (GTEx)
Human skeletal muscle transcriptomics (HPA)
Human skeletal muscle proteomics
HSPB1
HSPB2
HSPB3
HSPB7
HSPB8
MYOG
HSPB4
HSPB5
HSPB6
HSPB9
FC to cycling
Cycling
Diff.
p = 0.05
p = 0.04
p = 0.04
p = 0.02
p = 0.03
p = 0.02
n.s.
MYH3
MYOM1
HSPB7
UNC45B
Relative mRNA levels (AU)
D0
Myo
Res
p = 0.004
p = 0.001
n.s.

Supplementary Figure 5. Chaperone expression in skeletal muscle tissue is consistent across experimental systems and evolutionary conserved.

a. The correlation between the median transcript levels and median protein levels of 164 chaperones (top) and 10,129 protein-coding genes (bottom) in human skeletal muscle (r=0.61 and 0.37, respectively, p=2.2E-16 in both, Pearson correlation). Human proteins were associated with their median raw protein abundance across skeletal muscle samples[68].

b. The correlation between the differential protein levels of mouse myotubes versus undifferentiated C2C12 cells, and the transcript levels of their human homologous genes in skeletal muscle according to the HPA. Top: 65 chaperones were included (r=0.25, p=0.045, Pearson correlation). Bottom: 1,561 proteins were included (r=0.36, p=2.2E-16, Pearson correlation).

c. The correlation between the differential protein levels of mouse myotubes versus undifferentiated C2C12 cells, and the protein levels of their homologous proteins in human skeletal muscle[68]. Top: 65 chaperones (r=0.2, p=0.11, Pearson correlation). Bottom: 1,561 proteins (r=0.37, p<2.2E-16, Pearson correlation).

d. Expression of sHSP in human cycling and differentiating myoblast cell line (LHCN-M2). Total RNA was extracted from human myoblasts (LHCN-M2) that were proliferating (cycling) or differentiated for 5 days (5d). The expression levels of HSPB1-10 and myogenin (MYOG) were analyzed by RT-qPCR. Quantification is represented as fold-change to cycling condition (Ctrl) of ΔΔCt normalized for RPL0; n = 3 replicates. Data is presented as boxplot (center line, median; whiskers, minimum and maximum). P values were computed by using two-sided student's t-test. n.s. = non-significant. HSPB10 was not detected in neither cycling nor differentiating LHCN-M2 cells.

e. Expression of differentiation gene markers in C2C12 myoblast cell line. Total RNA was extracted from C2C12 myoblast cell line before (D0) or after maximal differentiation (Myo) or undifferentiated reserve cell (Res). The expression levels of MYH3, MYOM1, HSPB7 and UNC45B were analyzed by RT-qPCR. Quantification is represented as AU of ΔΔCt normalized for HPRT1. Data is presented as mean ± SE. P values were computed by using two-sided MW test, comparing to D0. The number of replicates n, for each condition is indicated.

Source data are provided as a Source Data file.

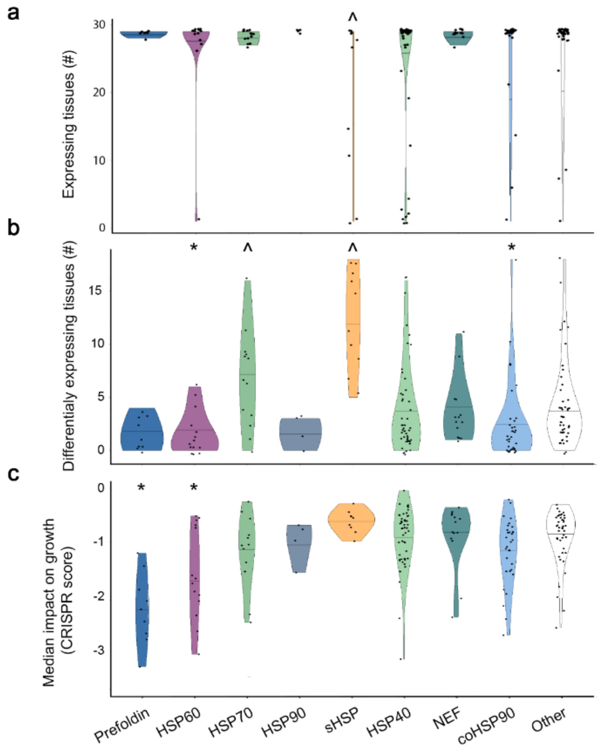

Supplementary Figure 6: The heterogeneous expression and essentiality patterns of chaperone families.

a. The number of tissues expressing a chaperone, per family. A chaperone was considered as expressed in a tissue if its expression level was ≥ 1 TPM. sHSPs were more tissue-specific than other families (adjusted *p*=0.027, one-sided MW test, marked with ^). The number of chaperones per family, from left to right: n=10, 14, 13, 4, 10, 49, 14, 34, 46.

b. The number of tissues in which a chaperone was differentially expressed (absolute (log_2fc)>1 and adjusted *p*<0.05) per family. HSP60s and coHSP90s were expressed more uniformly than other families (adjusted *p*=0.019 and *p*=0.011, MW test, marked with *), whereas sHSPs and HSP70s were more variably expressed than other families (adjusted *p*=2E-5 and *p*=0.033, respectively, one-sided MW test, marked with ^). The number of chaperones per family appears above.

c. The impact on growth of chaperones per family. For each chaperone, impact on growth was set to its minimal CRISPR score measured in 769 cell lines by the DepMap project. Chaperones' impact on growth was highly diverse in most chaperone families. Prefoldins and HSP60s were more important for growth than other families (adjusted *p*=1.5E-4 and *p*=0.045, respectively, one-sided MW test, marked with *). The number of chaperones per family, from left to right: n=9, 14, 12, 4, 9, 48, 14, 31, 45.

The middle line of every violin plot represents the median.

Source data are provided as a Source Data file.

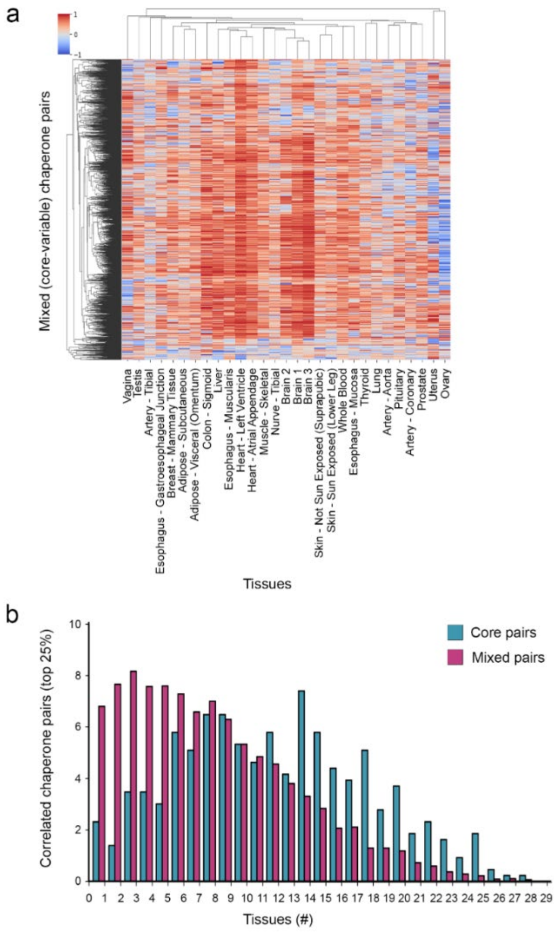

Supplementary Figure 7. Chaperone functional relationships differ between tissues.

a. A heatmap of the expression correlations between core and variable chaperones. Each row corresponds to a distinct mixed pair (composed of a core and variable chaperone), and shows its normalized expression correlation values across tissues. Across tissues, core chaperones modulated their relationships with variable chaperones.

b. The distribution of pairs of core chaperones (blue) and mixed pairs of core and variable chaperones (pink) by the number of tissues in which their expression levels were correlated. Pairs were considered correlated in a tissue if their expression correlation value was at the top 25% of the correlation values between chaperone pairs for that tissue. Pairs of core chaperones tend to be correlated across more tissues than mixed pairs (p=2.6E-7, two-sided KS test).

Source data are provided as a Source Data file.

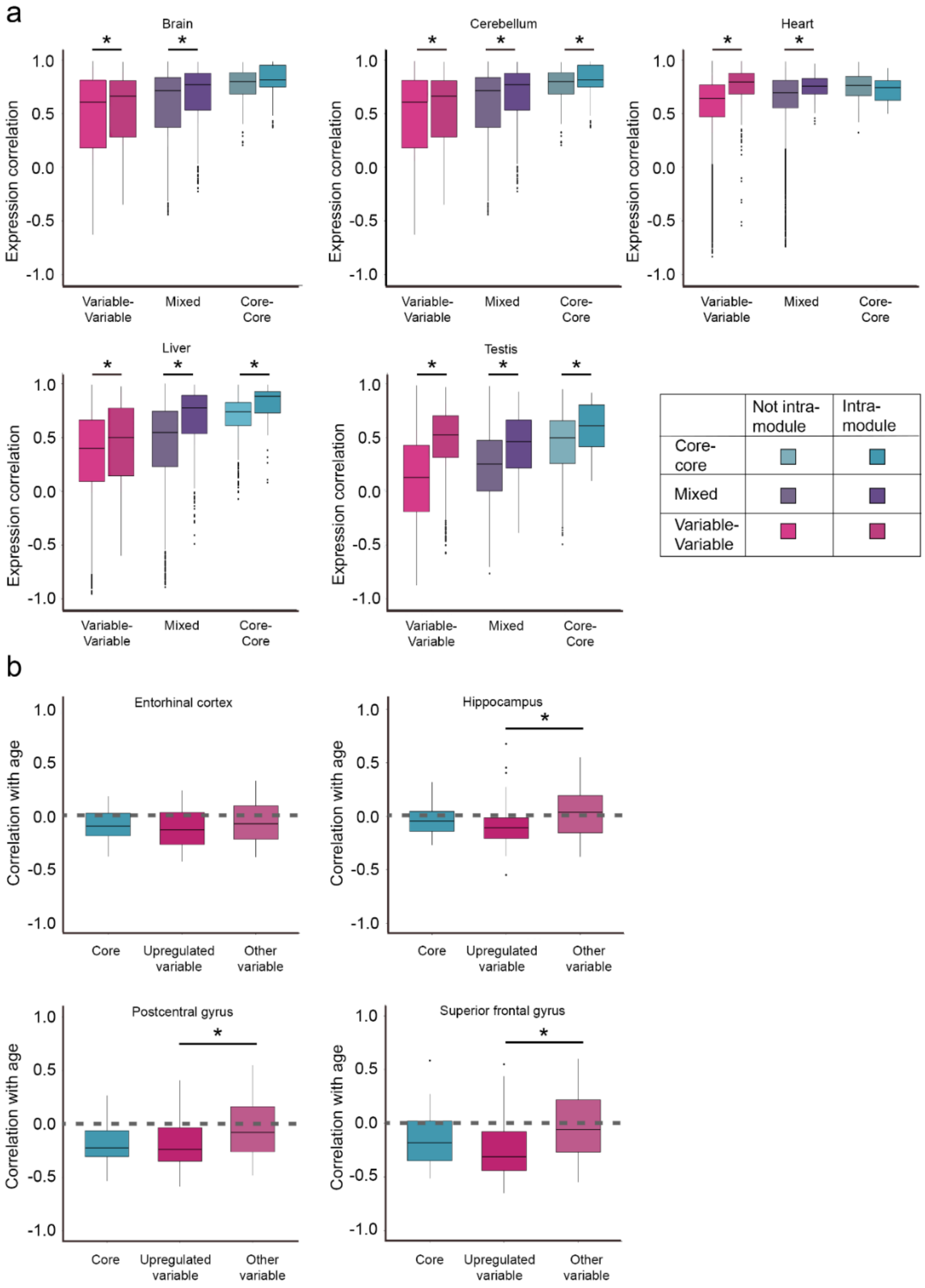

Supplementary Figure 8. Chaperone organization in organ development and brain aging.

a. Expression correlation values in adult tissues for chaperone pairs whose pair-mates share, or do not share, a developmental module. Per organ, chaperone pairs were divided into core-core (CC), mixed (core-variable), and variable-variable (VV) pairs. Per organ and subset, pairs belonging to the same developmental module were generally more highly correlated in adult tissues, and correlation values typically increased upon moving from VV to mixed to CC pairs. P-values were computed by using the

MW one-sided test and adjusted for multiple comparisons with Benjamini Hochberg correction. Adjusted p-values per organ and subset were as follows: Brain: VV 7.75E-10, mixed 2.99E-5, CC 0.086; Cerebellum: VV 0.014, mixed 4.46E-7, CC 0.013; Heart: VV 6.26E-75, mixed 1.2E-10, CC non-significant; Liver: VV 3E-6, mixed 1.4E-21, CC 2.99E-5; Testis: VV 2.58E-216, mixed 1.07E-31, CC 0.031. VV pairs n=12,090; mixed pairs in brain, cerebellum, liver and testis n=6,166; mixed pairs in heart n=5,973; CC pairs n=465.

b. The correlation between chaperone expression levels in distinct brain regions and age is shown for core chaperones (n=26), variable chaperones that were upregulated in adult brain (n=78), and variable chaperones that were not upregulated in brain (n=73, other). Expression levels of core and brain-upregulated chaperones decreased with age, whereas expression levels of remaining variable chaperones did not change. Differences between the two subsets of variable chaperones were generally statistically significant (entorhinal cortex p=0.047; hippocampus p=0.0003; postcentral gyrus p=0.0008; superior frontal gyrus p=6.4E-6; one-sided MW test).

In the boxplot representation, center line, median; box limits, upper and lower quartiles; whiskers, 1.5x interquartile range; points, outliers. Source data are provided as a Source Data file.

Supplementary References

1 Barral, J. M., Hutagalung, A. H., Brinker, A., Hartl, F. U. & Epstein, H. F. Role of the myosin assembly protein UNC-45 as a molecular chaperone for myosin. *Science* **295**, 669-671 (2002).

2 Price, M. G., Landsverk, M. L., Barral, J. M. & Epstein, H. F. Two mammalian UNC-45 isoforms are related to distinct cytoskeletal and muscle-specific functions. *J Cell Sci* **115**, 4013-4023 (2002).

3 Sugiyama, Y. *et al.* Muscle develops a specific form of small heat shock protein complex composed of MKBP/HSPB2 and HSPB3 during myogenic differentiation. *J Biol Chem* **275**, 1095-1104 (2000).

4 Morelli, F. F. *et al.* Aberrant Compartment Formation by HSPB2 Mislocalizes Lamin A and Compromises Nuclear Integrity and Function. *Cell Rep* **20**, 2100-2115, doi:10.1016/j.celrep.2017.08.018 (2017).

5 Mercer, E. J., Lin, Y. F., Cohen-Gould, L. & Evans, T. Hspb7 is a cardioprotective chaperone facilitating sarcomeric proteostasis. *Dev Biol* **435**, 41-55, doi:10.1016/j.ydbio.2018.01.005 (2018).

6 Wu, T. *et al.* HSPB7 is indispensable for heart development by modulating actin filament assembly. *Proc Natl Acad Sci U S A* **114**, 11956-11961, doi:10.1073/pnas.1713763114 (2017).

7 Juo, L. Y. *et al.* HSPB7 interacts with dimerized FLNC and its absence results in progressive myopathy in skeletal muscles. *J Cell Sci* **129**, 1661-1670, doi:10.1242/jcs.179887 (2016).

8 Tobin, S. W. *et al.* Regulation of Hspb7 by MEF2 and AP-1: implications for Hspb7 in muscle atrophy. *J Cell Sci* **129**, 4076-4090, doi:10.1242/jcs.190009 (2016).

9 Gardner, G. T. *et al.* Phosphorylation of Hsp20 Promotes Fibrotic Remodeling and Heart Failure. *JACC Basic Transl Sci* **4**, 188-199, doi:10.1016/j.jacbts.2018.11.007 (2019).

10 Dreiza, C. M. *et al.* The small heat shock protein, HSPB6, in muscle function and disease. *Cell Stress Chaperones* **15**, 1-11, doi:10.1007/s12192-009-0127-8 (2010).

11 Dimauro, I., Antonioni, A., Mercatelli, N. & Caporossi, D. The role of alphaB-crystallin in skeletal and cardiac muscle tissues. *Cell Stress Chaperones* **23**, 491-505, doi:10.1007/s12192-017-0866-x (2018).

12 Al-Tahan, S. *et al.* New family with HSPB8-associated autosomal dominant rimmed vacuolar myopathy. *Neurol Genet* **5**, e349, doi:10.1212/NXG.0000000000000349 (2019).

13 Ghaoui, R. *et al.* Mutations in HSPB8 causing a new phenotype of distal myopathy and motor neuropathy. *Neurology* **86**, 391-398, doi:10.1212/WNL.0000000000002324 (2016).

14 Nicolau, S., Liewluck, T., Elliott, J. L., Engel, A. G. & Milone, M. A novel heterozygous mutation in the C-terminal region of HSPB8 leads to limb-girdle rimmed vacuolar myopathy. *Neuromuscul Disord* **30**, 236-240, doi:10.1016/j.nmd.2020.02.005 (2020).

15 Arndt, V. *et al.* Chaperone-assisted selective autophagy is essential for muscle maintenance. *Curr Biol* **20**, 143-148, doi:10.1016/j.cub.2009.11.022 (2010).

16 Cristofani, R. *et al.* The Regulation of the Small Heat Shock Protein B8 in Misfolding Protein Diseases Causing Motoneuronal and Muscle Cell Death. *Front Neurosci* **13**, 796, doi:10.3389/fnins.2019.00796 (2019).

17 Kathage, B. *et al.* The cochaperone BAG3 coordinates protein synthesis and autophagy under mechanical strain through spatial regulation of mTORC1. *Biochim Biophys Acta Mol Cell Res* **1864**, 62-75, doi:10.1016/j.bbamcr.2016.10.007 (2017).

18 Tucker, N. R. & Shelden, E. A. Hsp27 associates with the titin filament system in heat-shocked zebrafish cardiomyocytes. *Exp Cell Res* **315**, 3176-3186, doi:10.1016/j.yexcr.2009.06.030 (2009).

19 Ago, T. *et al.* A redox-dependent pathway for regulating class II HDACs and cardiac hypertrophy. *Cell* **133**, 978-993, doi:10.1016/j.cell.2008.04.041 (2008).

20 Linnstaedt, S. D. *et al.* A Functional riboSNitch in the 3' Untranslated Region of FKBP5 Alters MicroRNA-320a Binding Efficiency and Mediates Vulnerability to Chronic Post-Traumatic Pain. *J Neurosci* **38**, 8407-8420, doi:10.1523/JNEUROSCI.3458-17.2018 (2018).

21 Balsevich, G. *et al.* Stress-responsive FKBP51 regulates AKT2-AS160 signaling and metabolic function. *Nat Commun* **8**, 1725, doi:10.1038/s41467-017-01783-y (2017).
22 Birket, M. J. *et al.* A Human Stem Cell Model of Fabry Disease Implicates LIMP-2 Accumulation in Cardiomyocyte Pathology. *Stem Cell Reports* **13**, 380-393, doi:10.1016/j.stemcr.2019.07.004 (2019).
23 Dorsch, L. M. *et al.* Protein Quality Control Activation and Microtubule Remodeling in Hypertrophic Cardiomyopathy. *Cells* **8**, doi:10.3390/cells8070741 (2019).
24 Ferrer-Martinez, A. *et al.* Long-term cultured human myotubes decrease contractile gene expression and regulate apoptosis-related genes. *Gene* **384**, 145-153, doi:10.1016/j.gene.2006.07.042 (2006).
25 Abdul, K. M., Terada, K., Gotoh, T., Hafizur, R. M. & Mori, M. Characterization and functional analysis of a heart-enriched DnaJ/ Hsp40 homolog dj4/DjA4. *Cell Stress Chaperones* **7**, 156-166, doi:10.1379/1466-1268(2002)007<0156:cafaoa>2.0.co;2 (2002).
26 Sarparanta, J. *et al.* Mutations affecting the cytoplasmic functions of the co-chaperone DNAJB6 cause limb-girdle muscular dystrophy. *Nat Genet* **44**, 450-455, S451-452 (2012).
27 Huang, H. *et al.* Identification of Potential Gene Interactions in Heart Failure Caused by Idiopathic Dilated Cardiomyopathy. *Med Sci Monit* **24**, 7697-7709, doi:10.12659/MSM.912984 (2018).
28 Shiffman, D. *et al.* Genome-wide study of gene variants associated with differential cardiovascular event reduction by pravastatin therapy. *PLoS One* **7**, e38240, doi:10.1371/journal.pone.0038240 (2012).
29 Hayashi, M. *et al.* A crucial role of mitochondrial Hsp40 in preventing dilated cardiomyopathy. *Nat Med* **12**, 128-132, doi:10.1038/nm1327 (2006).
30 Cheng, L. H. *et al.* Mitochondrial co-chaperone protein Tid1 is required for energy homeostasis during skeletal myogenesis. *Stem Cell Res Ther* **7**, 185, doi:10.1186/s13287-016-0443-8 (2016).
31 Chao, C. N. *et al.* Tid1-S attenuates LPS-induced cardiac hypertrophy and apoptosis through ER-a mediated modulation of p-PI3K/p-Akt signaling cascade. *J Cell Biochem* **120**, 16703-16710, doi:10.1002/jcb.28928 (2019).
32 Bernardi, P. & Bonaldo, P. Mitochondrial dysfunction and defective autophagy in the pathogenesis of collagen VI muscular dystrophies. *Cold Spring Harb Perspect Biol* **5**, a011387, doi:10.1101/cshperspect.a011387 (2013).
33 Zhang, P., Lu, Y., Yu, D., Zhang, D. & Hu, W. TRAP1 Provides Protection Against Myocardial Ischemia-Reperfusion Injury by Ameliorating Mitochondrial Dysfunction. *Cell Physiol Biochem* **36**, 2072-2082, doi:10.1159/000430174 (2015).
34 Zhou, T. T. *et al.* Mitochondrial Translocation of DJ-1 Is Mediated by Grp75: Implication in Cardioprotection of Resveratrol Against Hypoxia/Reoxygenation-Induced Oxidative Stress. *J Cardiovasc Pharmacol* **75**, 305-313, doi:10.1097/FJC.0000000000000805 (2020).
35 Thakur, S. S., Swiderski, K., Ryall, J. G. & Lynch, G. S. Therapeutic potential of heat shock protein induction for muscular dystrophy and other muscle wasting conditions. *Philos Trans R Soc Lond B Biol Sci* **373**, doi:10.1098/rstb.2016.0528 (2018).
36 Ornatsky, O. I., Connor, M. K. & Hood, D. A. Expression of stress proteins and mitochondrial chaperonins in chronically stimulated skeletal muscle. *Biochem J* **311 (Pt 1)**, 119-123, doi:10.1042/bj3110119 (1995).
37 Lim, D. S., Roberts, R. & Marian, A. J. Expression profiling of cardiac genes in human hypertrophic cardiomyopathy: insight into the pathogenesis of phenotypes. *J Am Coll Cardiol* **38**, 1175-1180, doi:10.1016/s0735-1097(01)01509-1 (2001).
38 Glazier, A. A. *et al.* HSC70 is a chaperone for wild-type and mutant cardiac myosin binding protein C. *JCI Insight* **3**, doi:10.1172/jci.insight.99319 (2018).
39 Davey, K. M. *et al.* Mutation of DNAJC19, a human homologue of yeast inner mitochondrial membrane co-chaperones, causes DCMA syndrome, a novel autosomal recessive Barth syndrome-like condition. *J Med Genet* **43**, 385-393, doi:10.1136/jmg.2005.036657 (2006).
40 Rohani, L. *et al.* Reversible Mitochondrial Fragmentation in iPSC-Derived Cardiomyocytes From Children With DCMA, a Mitochondrial Cardiomyopathy. *Can J Cardiol* **36**, 554-563, doi:10.1016/j.cjca.2019.09.021 (2020).

41 Arhzaouy, K. *et al.* VCP maintains lysosomal homeostasis and TFEB activity in differentiated skeletal muscle. *Autophagy* **15**, 1082-1099, doi:10.1080/15548627.2019.1569933 (2019).

42 Kustermann, M. *et al.* Loss of the novel Vcp (valosin containing protein) interactor Washc4 interferes with autophagy-mediated proteostasis in striated muscle and leads to myopathy in vivo. *Autophagy* **14**, 1911-1927, doi:10.1080/15548627.2018.1491491 (2018).

43 Lubelwana Hafver, T. *et al.* Mapping the in vitro interactome of cardiac sodium (Na(+))-calcium (Ca(2+)) exchanger 1 (NCX1). *Proteomics* **17**, doi:10.1002/pmic.201600417 (2017).

44 Li, J. *et al.* Tom70 serves as a molecular switch to determine pathological cardiac hypertrophy. *Cell Res* **24**, 977-993, doi:10.1038/cr.2014.94 (2014).

45 Escobedo, J., Pucci, A. M. & Koh, T. J. HSP25 protects skeletal muscle cells against oxidative stress. *Free Radic Biol Med* **37**, 1455-1462, doi:10.1016/j.freeradbiomed.2004.07.024 (2004).

46 Mohamed, B. A. *et al.* Targeted disruption of Hspa4 gene leads to cardiac hypertrophy and fibrosis. *J Mol Cell Cardiol* **53**, 459-468, doi:10.1016/j.yjmcc.2012.07.014 (2012).

47 Chang, E. 1,25-Dihydroxyvitamin D Decreases Tertiary Butyl-Hydrogen Peroxide-Induced Oxidative Stress and Increases AMPK/SIRT1 Activation in C2C12 Muscle Cells. *Molecules* **24**, doi:10.3390/molecules24213903 (2019).

48 Colak, D. *et al.* Integrated Left Ventricular Global Transcriptome and Proteome Profiling in Human End-Stage Dilated Cardiomyopathy. *PLoS One* **11**, e0162669, doi:10.1371/journal.pone.0162669 (2016).

49 Peng, Y. J. *et al.* Regulation of CLC-1 chloride channel biosynthesis by FKBP8 and Hsp90beta. *Sci Rep* **6**, 32444, doi:10.1038/srep32444 (2016).

50 Muraguchi, T., Kawawa, A. & Kubota, S. Prohibitin protects against hypoxia-induced H9c2 cardiomyocyte cell death. *Biomed Res* **31**, 113-122, doi:10.2220/biomedres.31.113 (2010).

51 Liu, X. *et al.* Prohibitin protects against oxidative stress-induced cell injury in cultured neonatal cardiomyocyte. *Cell Stress Chaperones* **14**, 311-319, doi:10.1007/s12192-008-0086-5 (2009).

52 Wattin, M. *et al.* Modulation of Protein Quality Control and Proteasome to Autophagy Switch in Immortalized Myoblasts from Duchenne Muscular Dystrophy Patients. *Int J Mol Sci* **19**, doi:10.3390/ijms19010178 (2018).

53 Rusmini, P. *et al.* Aberrant Autophagic Response in The Muscle of A Knock-in Mouse Model of Spinal and Bulbar Muscular Atrophy. *Sci Rep* **5**, 15174, doi:10.1038/srep15174 (2015).

54 Crippa, V. *et al.* Differential autophagy power in the spinal cord and muscle of transgenic ALS mice. *Front Cell Neurosci* **7**, 234, doi:10.3389/fncel.2013.00234 (2013).

55 Sun, L., Liu, L., Yang, X. J. & Wu, Z. Akt binds prohibitin 2 and relieves its repression of MyoD and muscle differentiation. *J Cell Sci* **117**, 3021-3029, doi:10.1242/jcs.01142 (2004).

56 Sun, L. *et al.* CaMK IV phosphorylates prohibitin 2 and regulates prohibitin 2-mediated repression of MEF2 transcription. *Cell Signal* **23**, 1686-1690, doi:10.1016/j.cellsig.2011.06.005 (2011).

57 Saleh, A., Subramaniam, G., Raychaudhuri, S. & Dhawan, J. Cytoplasmic sequestration of the RhoA effector mDiaphanous1 by Prohibitin2 promotes muscle differentiation. *Sci Rep* **9**, 8302, doi:10.1038/s41598-019-44749-4 (2019).

58 Vang, S. *et al.* Actin mutations in hypertrophic and dilated cardiomyopathy cause inefficient protein folding and perturbed filament formation. *FEBS J* **272**, 2037-2049 (2005).

59 Ghosh, A., Dai, Y., Biswas, P. & Stuehr, D. J. Myoglobin maturation is driven by the hsp90 chaperone machinery and by soluble guanylyl cyclase. *FASEB J* **33**, 9885-9896, doi:10.1096/fj.201802793RR (2019).

60 Yun, B. G. & Matts, R. L. Hsp90 functions to balance the phosphorylation state of Akt during C2C12 myoblast differentiation. *Cell Signal* **17**, 1477-1485, doi:10.1016/j.cellsig.2005.03.006 (2005).

61 Yun, B. G. & Matts, R. L. Differential effects of Hsp90 inhibition on protein kinases regulating signal transduction pathways required for myoblast differentiation. *Exp Cell Res* **307**, 212-223, doi:10.1016/j.yexcr.2005.03.003 (2005).

62 Ranek, M. J., Stachowski, M. J., Kirk, J. A. & Willis, M. S. The role of heat shock proteins and co-chaperones in heart failure. *Philos Trans R Soc Lond B Biol Sci* **373**, doi:10.1098/rstb.2016.0530 (2018).
63 Willis, M. S. *et al.* Carboxyl terminus of Hsp70-interacting protein (CHIP) is required to modulate cardiac hypertrophy and attenuate autophagy during exercise. *Cell Biochem Funct* **31**, 724-735, doi:10.1002/cbf.2962 (2013).
64 Schisler, J. C., Patterson, C. & Willis, M. S. Skeletal Muscle Mitochondrial Alterations in Carboxyl Terminus of Hsc70 Interacting Protein (Chip) -/- Mice. *Afr J Cell Pathol* **6**, 28-36 (2016).
65 Yang, K. *et al.* Carboxyl terminus of heat shock protein 70-interacting protein inhibits angiotensin II-induced cardiac remodeling. *Am J Hypertens* **25**, 994-1001, doi:10.1038/ajh.2012.74 (2012).
66 Diofano, F. *et al.* Genetic compensation prevents myopathy and heart failure in an in vivo model of Bag3 deficiency. *PLoS Genet* **16**, e1009088, doi:10.1371/journal.pgen.1009088 (2020).
67 Rahmani, P., Rogalski, T. & Moerman, D. G. The C. elegans UNC-23 protein, a member of the BCL-2-associated athanogene (BAG) family of chaperone regulators, interacts with HSP-1 to regulate cell attachment and maintain hypodermal integrity. *Worm* **4**, e1023496, doi:10.1080/21624054.2015.1023496 (2015).
68 Jiang, L. *et al.* A Quantitative Proteome Map of the Human Body. *Cell* **183**, 269-283 e219, doi:10.1016/j.cell.2020.08.036 (2020).
