# Supplementary material for: The landscape of molecular chaperones across human tissues reveals a layered architecture of core and variable chaperones
Source: Nat Commun. 2021 Apr 12;12:2180. doi: 10.1038/s41467-021-22369-9 (PMC8042005; doi:10.1038/s41467-021-22369-9)
Supplement: Supplementary file 2 — Description of Additional Supplementary Files [file 41467_2021_22369_MOESM2_ESM.docx]

**Description of Supplementary Files**

**File Name: Supplementary Data 1**

**Description:** Chaperones analyzed in the study.

**File Name: Supplementary Data 2**

**Description:** Tissues analyzed in the study.

**File Name: Supplementary Data 3**

**Description:** Disease-causing chaperones and affected tissues.

**File Name: Supplementary Data 4**

**Description:** Data of expression fold-change of chaperones across tissues.

**File Name: Supplementary Data 5**

**Description:** Data of pairwise chaperone co-expression correlations per tissue.

**File Name: Supplementary Data 6**

**Description:** The analyzed mass spectrometry proteomics data.
