# Supplementary material for: The landscape of molecular chaperones across human tissues reveals a layered architecture of core and variable chaperones
Source: Nat Commun. 2021 Apr 12;12:2180. doi: 10.1038/s41467-021-22369-9 (PMC8042005; doi:10.1038/s41467-021-22369-9)
Supplement: Supplementary file 9 — Reporting Summary [file 41467_2021_22369_MOESM9_ESM.pdf]

nature research

Corresponding author(s): Esti Yeger-Lotem

Last updated by author(s): 2/2/2021

Reporting Summary

Nature Research wishes to improve the reproducibility of the work that we publish. This form provides structure for consistency and transparency in reporting. For further information on Nature Research policies, see our Editorial Policies and the Editorial Policy Checklist.

Statistics

For all statistical analyses, confirm that the following items are present in the figure legend, table legend, main text, or Methods section.

n/a	Confirmed	
☐	☒	The exact sample size (*n*) for each experimental group/condition, given as a discrete number and unit of measurement
☐	☒	A statement on whether measurements were taken from distinct samples or whether the same sample was measured repeatedly
☐	☒	The statistical test(s) used AND whether they are one- or two-sided<br>*Only common tests should be described solely by name; describe more complex techniques in the Methods section.*
☒	☐	A description of all covariates tested
☐	☒	A description of any assumptions or corrections, such as tests of normality and adjustment for multiple comparisons
☐	☒	A full description of the statistical parameters including central tendency (e.g. means) or other basic estimates (e.g. regression coefficient) AND variation (e.g. standard deviation) or associated estimates of uncertainty (e.g. confidence intervals)
☐	☒	For null hypothesis testing, the test statistic (e.g. *F*, *t*, *r*) with confidence intervals, effect sizes, degrees of freedom and *P* value noted<br>*Give P values as exact values whenever suitable.*
☒	☐	For Bayesian analysis, information on the choice of priors and Markov chain Monte Carlo settings
☒	☐	For hierarchical and complex designs, identification of the appropriate level for tests and full reporting of outcomes
☐	☒	Estimates of effect sizes (e.g. Cohen's *d*, Pearson's *r*), indicating how they were calculated

Our web collection on statistics for biologists contains articles on many of the points above.

Software and code

Policy information about availability of computer code

Data collection	MaxQuant 1.5.2.8;
Data analysis	BioMart; Limma 3.34.5; edgeR 3.20.6; cytoscape.js plugin 3.2.2; OrthoList2 tool; numpy 1.8.2; scipy 0.13.3

For manuscripts utilizing custom algorithms or software that are central to the research but not yet described in published literature, software must be made available to editors and reviewers. We strongly encourage code deposition in a community repository (e.g. GitHub). See the Nature Research guidelines for submitting code & software for further information.

Data

Policy information about availability of data

All manuscripts must include a data availability statement. This statement should provide the following information, where applicable:

- Accession codes, unique identifiers, or web links for publicly available datasets
- A list of figures that have associated raw data
- A description of any restrictions on data availability

Data of chaperones analyzed in the study are available as Supplementary Data 1. Data of tissues analyzed in the study appear in Supplementary Data 2. Data of disease-associated chaperones appear in Supplementary Data 3. Data of expression fold-change of chaperones across tissues is available as Supplementary Data 4. Data of pairwise chaperone co-expression correlations per tissue are available as Supplementary Data 5, and through the ChaperoneNet webtool (https://netbio.bgu.ac.il/chapnet/). The mass spectrometry proteomics data were deposited in ProteomeXchange Consortium via the PRIDE103 partner repository with the dataset identifier PXD022678 (LC-MS/MS of C2C12 mouse myoblast cell line) [http://proteomecentral.proteomexchange.org/cgi/GetDataset?ID=PXD022678]. The analyzed mass spectrometry proteomics data of mouse myoblasts are available as Supplementary Data 6. Source data for Figures 1-6 are provided with this paper. The following databases were used in the study: GTEx v7 http://www.gtexportal.org/home/; The Human Protein Atlas http://www.proteinatlas.org; Mouse Genome Informatics http://www.informatics.jax.org/; BioMart http://m.ensembl.org/biomart/martview; OMIM http://www.omim.org/; and DepMap http://depmap.org.

Field-specific reporting

Please select the one below that is the best fit for your research. If you are not sure, read the appropriate sections before making your selection.

- [x] Life sciences
- [ ] Behavioural & social sciences
- [ ] Ecological, evolutionary & environmental sciences

For a reference copy of the document with all sections, see nature.com/documents/nr-reporting-summary-flat.pdf

Life sciences study design

All studies must disclose on these points even when the disclosure is negative.

Sample size	No sample size calculation were made. At least 3 biologic repeats were used to enable us to calculate statistical significance. Our experiments meet the established standards of the field.
Data exclusions	LC-MS/MS were analyzed using MaxQuant 1.5.2.8 versus Mus musculus part of the Uniprot database and known contaminants were removed Only proteins that were identified with at least 2 peptides were tested for significant differences (q-value < 0.05).
Replication	Each experimental condition used included at least 3 biologic repeats. For qPCR data 3 technical replicates were also included. All attempts at replication were successful.
Randomization	Randomization was not relevant to this study as there was no possibility of bias in the experiments performed. All Samples were collected as indicated in the Method section and samples were analyzed using the same procedures.
Blinding	Blinding is not relevant to this study as the readouts for all experiments were not subjective. LC-MS/MS samples were analyzed by a mass spec facility (Technion, Israel). sHSP verification experiment was done by Prof. Carra group without prior information on which genes were identified as upregulated by the computational analysis.

Reporting for specific materials, systems and methods

We require information from authors about some types of materials, experimental systems and methods used in many studies. Here, indicate whether each material, system or method listed is relevant to your study. If you are not sure if a list item applies to your research, read the appropriate section before selecting a response.

Materials & experimental systems

n/a	Involved in the study	
x		Antibodies
	x	Eukaryotic cell lines
x		Palaeontology and archaeology
x		Animals and other organisms
x		Human research participants
x		Clinical data
x		Dual use research of concern

Methods

n/a	Involved in the study	
x		ChIP-seq
x		Flow cytometry
x		MRI-based neuroimaging

Eukaryotic cell lines

Policy information about cell lines

Cell line source(s)	C2C12 (ATCC CRL1772); LHCN-M2 (RRID:CVCL_8890) were provided by Dr. Elena Pegoraro and were previously described (Morelli et al., Cell Reports 2017).
Authentication	Morphology check by microscope
Mycoplasma contamination	Cells were routinely tested for mycoplasma contamination. All cell lines tested negative for mycoplasma contamination.
Commonly misidentified lines (See ICLAC register)	No commonly misidentified cell lines were used in the study.
